# Multi-omics investigation reveals unique markers in *Klebsiella pneumoniae* compared to closely related species

**DOI:** 10.3389/fmicb.2025.1657680

**Published:** 2025-08-20

**Authors:** Lena-Sophie Swiatek, Kristin Surmann, Elias Eger, Justus U. Müller, Manuela Gesell Salazar, Stefan E. Heiden, Guido Werner, Nils-Olaf Hübner, Jürgen A. Bohnert, Karsten Becker, Uwe Völker, Michael Schwabe, Katharina Schaufler

**Affiliations:** ^1^Department of Epidemiology and Ecology of Antimicrobial Resistance, Helmholtz Institute for One Health, Helmholtz Centre for Infection Research HZI, Greifswald, Germany; ^2^Department of Functional Genomics, Interfaculty Institute for Genetics and Functional Genomics, University Medicine Greifswald, Greifswald, Germany; ^3^Division of Nosocomial Pathogens and Antibiotic Resistances, Department of Infectious Diseases, Robert Koch Institute, Wernigerode, Germany; ^4^Central Unit for Infection Prevention and Control, University Medicine Greifswald, Greifswald, Germany; ^5^Friedrich Loeffler-Institute of Medical Microbiology, University Medicine Greifswald, Greifswald, Germany; ^6^University Medicine Greifswald, Greifswald, Germany

**Keywords:** multi-omics, *Klebsiella pneumoniae* species complex and its differences, multidrug resistance, markers, synthetic human urine

## Abstract

**Introduction:**

The *Klebsiella pneumoniae* (KP) species complex (KpSC) comprises KP as the predominant species, and six other taxa including two subspecies each of *Klebsiella* var*iicola* (KV) and *Klebsiella quasipneumoniae* (KQ), all capable of causing clinical infections and often challenging to differentiate. Among these, KP is by far the most clinically significant, with the emergence of multidrug-resistant and hypervirulent strains leading to severe infections and limited treatment options, underscoring the need to understand the genomic features of KP.

**Methods:**

This study compared globally disseminated KP lineages with less abundant KV strains in synthetic human urine (SHU) across multiple omics levels to identify characteristics differentiating these closely related species. Moreover, a large genomic dataset of over 6,000 publicly available genomes of KP, KV, and KQ was constructed for comparisons to other members of the KpSC.

**Results:**

Among eight clinical KP strains representing four different sequence types (STs), we identified 107 genes comprising the KP-specific core genome, while these genes were absent from two selected KV genomes. Transcriptome and proteome analyses in SHU revealed different regulatory patterns between KP and KV strains, with metabolic responses playing a pivotal role. A total of 193 genes specific to the investigated KP STs were identified, exhibiting differential expression at the transcriptomic and/or proteomic levels. Comparison to the genomic dataset highlighted genes adaptively regulated or uniquely present in KP genomes. For example, certain genes for citrate metabolism are uniquely upregulated in KP and a gene cluster for the cellobiose phosphotransferase system, previously linked to bacterial virulence and biofilm formation, was found exclusively in KP.

**Discussion:**

Our study underscores the metabolic flexibility of KP strains in response to specific environmental conditions, potentially contributing to opportunistic pathogenicity. We identified markers enriched in KP STs, providing a foundation for future investigations including their relevance for diagnostics.

## 1 Introduction

The genus *Klebsiella* encompasses diverse opportunistic pathogens, which are either part of the *Klebsiella pneumoniae* species complex (KpSC), sharing >95.0% average nucleotide identity, or belonging to other *Klebsiella* species (e.g., *K. oxytoca, K. michiganensis* or *K. pasteurii*) (Wyres et al., 2020a). The KpSC consists of seven taxa, including *K. pneumoniae* (KP), and the subspecies of *K. quasipneumoniae*, namely *K. quasipneumoniae* subsp. *quasipneumoniae* and *K. quasipneumoniae* subsp. *similipneumoniae* (summarized as KQ), and *K.* var*iicola*, namely *Klebsiella variicola* subsp. *variicola* and *Klebsiella variicola* subsp. *tropica* (summarized as KV), which are closely related (Rosenblueth et al., 2004; Brisse et al., 2014; Holt et al., 2015). Although KQ and KV have the potential to cause clinical outbreaks, they are generally less prevalent in clinical settings compared to KP (Brisse and Verhoef, 2001; Holt et al., 2015; Wyres et al., 2019, 2020b; Lam et al., 2021; Gorrie et al., 2022; Kochan et al., 2022; Thorpe et al., 2022; Calland et al., 2023). Due to their similarity, differentiation using microbiological methods or MALDI-TOF is often inaccurate (Wyres et al., 2020b; Gorrie et al., 2022). However, local whole-genome sequencing (WGS) investigations of clinical isolates in regions such as Australia, South and Southeast Asia, and Italy have shown that 82.0–91.0% of isolates previously identified as KpSC are actually KP, 2.5–14.0% are KV, and 4.0–6.9% are KQ, with other *Klebsiella* species occurring even less frequently (Wyres et al., 2020b; Gorrie et al., 2022; Calland et al., 2023). The dominant prevalence of KP within the KpSC is evident not only in clinical samples but also across various environmental niches including farm animal, community members, hospital patients, water and other environmental sources (65.0% KP, 24.0% KQ, 6.0% KV, and 4.0% *K. aerogenes* (Calland et al., 2023)). Another study in Italy demonstrated that KP from clinical, community, animal and environmental niches accounts for 49.0%, followed by *K. michiganensis* (9.0%), KV (8.5%), and KQ (1.0%) of all investigated isolates (Thorpe et al., 2022). In 2021, Lam et al. reported that genomic data for *Klebsiella* spp. (*n* = 13,156) is mostly available for KP (86.0% of genomes), followed by other members of the KpSC (9.4%) (Lam et al., 2021). Moreover, this large-scale genomic analysis showed that the frequencies of harboring crucial virulence factors is notably lower in KQ (0.8–2.0%) and KV (0.4–3.0%) compared to KP (7.0–44.0%) (Lam et al., 2021). In line with this, other studies have consistently shown that yersiniabactin siderophores and the virulence-associated regulator of the mucoid phenotype *rmpA*, were detected in every third KP isolate in Australia (Gorrie et al., 2022) and Japan (Imai et al., 2019; Matsuda et al., 2023), but were rarely found in KV and KQ (Lu et al., 2018). Similarly, the highest carriage of antibiotic resistance genes occurred among clinical KP isolates from Australia and Italy (21.0%) (Gorrie et al., 2022) compared to 7.0–8.0% (Gorrie et al., 2022) in isolates identified as KV and KQ (Gorrie et al., 2022; Thorpe et al., 2022). Additionally, the predominant occurrence of these genes in KP was confirmed in a non-redundant *Klebsiella* dataset of 9,705 genomes (Lam et al., 2021).

A recent systematic analysis of the global burden of bacterial antimicrobial resistance (AMR) estimated the significant public health impact of multidrug-resistant (MDR) KP infections, which were associated with over 600,000 deaths worldwide in 2019 (Murray et al., 2022). This is further emphasized by the World Health Organization’s Bacterial Priority Pathogens List in 2024, which ranks carbapenem- and third-generation cephalosporin-resistant KP among the top ten critical pathogens (Tacconelli et al., 2018; World Health Organization, 2024). KP colonizes human mucosal surfaces, serving as important reservoirs for subsequent infection (Davis and Matsen, 1974; Gorrie et al., 2017, 2022; Martin and Bachman, 2018; Chang et al., 2021). As an opportunistic pathogen, KP is associated with a wide range of diseases, including pneumonia, urinary tract infection (UTI), pyogenic liver abscess, meningitis, and bloodstream infections (Friedländer, 1882; Podschun and Ullmann, 1998; Chang et al., 2000; Ko et al., 2002; Magill et al., 2014). Multiple studies have identified the urinary tract as the most frequent isolation source of KP, with prevalence ranging from 39.0–66.0% (Gorrie et al., 2022; Matsuda et al., 2023; Rodríguez-Medina et al., 2024), and have recognized KP as the second most common cause of UTI after *Escherichia coli* (Flores-Mireles et al., 2015).

Multi-locus sequence typing (MLST), applicable across the entirety of KpSC (Bialek-Davenet et al., 2014), facilitates the differentiation of KP into various sequence types (STs), each distinguished by unique global dissemination and distribution patterns (Diancourt et al., 2005; Wyres et al., 2020a). The aforementioned study by Lam et al. revealed considerable phylogenetic diversity, encompassing isolates belonging to at least 1,452 distinct STs (Lam et al., 2021). Among a selected non-redundant KP-dataset, MDR and hypervirulent clinical isolates comprised 63.4% and were distributed among 30 different STs (Lam et al., 2021).

The most successful STs, also termed international high-risk clonal lineages, exhibit widespread dissemination globally and are primarily associated with KP. They contribute significantly to the propagation of AMR genes, often harboring them on extra-chromosomal elements. These lineages possess notable colonization and persistence capabilities within hosts, coupled with heightened virulence and pathogenicity, resulting in recurrent infections (Mathers et al., 2015). However, the mechanisms underlying their global dissemination potential remain inadequately explored (de Lagarde et al., 2021). Noteworthy among the high-risk clonal lineages are ST15, ST147, and ST258, primarily associated with MDR, including the expression of extended-spectrum *β*-lactamases (ESBL) and carbapenemases (Knothe et al., 1983; Nordmann et al., 2009; Wyres and Holt, 2018). Additionally, certain STs, such as ST23, are characterized mostly as hypervirulent *K. pneumoniae* (hvKp), marked by the presence of multiple virulence factors and a common lack of MDR. These strains are often associated with severe infections in healthy community members (Shon et al., 2013; Bialek-Davenet et al., 2014; Russo et al., 2018). Of particular concern is the emergence of convergent KP strains, combining traits of hvKp and MDR strains, exemplified by ST307 (Bialek-Davenet et al., 2014; Li et al., 2014; Gu et al., 2018; Heiden et al., 2020; Lam et al., 2021; Mendes et al., 2022; ECDC, 2024), which may even demonstrate resistance to recently approved antibiotics like cefiderocol (Schaufler et al., 2023). Local outbreaks featuring sporadic KP STs underscore the opportunistic nature and complexity of the KP species (Wyres et al., 2020a).

The “success” and prevalence of KP, particularly in clinical environments, is influenced by a multifaceted interplay of factors, including the accumulation of resistance and/or virulence genes, as well as colonization abilities, which are often prerequisites for subsequent infections (Gorrie et al., 2022). Despite KP’s prominent presence in clinical settings, investigating mechanisms beyond conventional virulence and resistance attributes is crutial (Moraes et al., 2024; O’Brien et al., 2025). Leveraging the available genomic data could facilitate more in-depth investigations into the distinctions between KP and other closely related species, particularly within the KpSC. The multifaceted diversity within the KpSC is evident in its complex accessory genome, which harbors a multitude of fitness and virulence factors associated with capsule and lipopolysaccharide production, iron acquisition, and various other functions (Holt et al., 2015). While gene content remains relatively conserved within lineages among different clinical isolates, significant diversity exists among different lineages, even within the same species (Gorrie et al., 2022).

Our hypothesis posits that KP displays unique characteristics distinct from closely related species such as KQ and KV. To explore this hypothesis, our study utilized genomics, transcriptomics, and proteomics analyses under conditions mimicking the urinary tract environment. We aimed to identify specific molecular markers that could elucidate differences within the complex that might also contribute to improved future diagnostics.

## 2 Materials and methods

### 2.1 Bacterial strains

We included four of the most prevalent KP sequence types (ST15, ST147, ST258, and ST307). Strains belonging to these KP STs are recognized for their MDR and/or virulence profiles (Supplementary Figure 1) (Wyres et al., 2020a; Lam et al., 2021). We specifically selected two clinical isolates for each of these STs, along with two strains of KV, for comprehensive transcriptomic, proteomic, and phenotypic analysis (Table 1). The decision to include KV for comparison was based on several factors: (i) epidemiological evidence suggests that KV infections are notably less frequent in clinical settings compared to KP (Brisse et al., 2014; Magill et al., 2014; Holt et al., 2015; Wyres et al., 2019; Gorrie et al., 2022; Thorpe et al., 2022; Matsuda et al., 2023), (ii) the close relationship within the KpSC allowed for a more comprehensive understanding of the differentiating factors (Holt et al., 2015; Wyres et al., 2019), and (iii) the availability of a sufficient number of KV genomes in public databases facilitated comparative genomic analyses to explore distinctions between KV and KP (Lam et al., 2021). Moreover, we selected KV instead of other KP STs because various KP STs often emerge as local problem clones within specific regions or healthcare settings (Wyres et al., 2020a). The KV isolates of the ST347 and ST906 were provided from a sampling set of clinical *Klebsiella* spp. isolates (Becker et al., 2018). The definition of different pathotypes of the isolates was done by using Kleborate to calculate the resistance score (0 = no ESBL, no carbapenemases (CARB) (regardless of colistin resistance); 1 = ESBL, no CARB (regardless of colistin resistance); 2 = CARB without colistin resistance (regardless of ESBL genes or OmpK mutations); 3 = CARB with colistin resistance (regardless of ESBL genes or OmpK mutations)) and the virulence score (0 = negative for all of yersiniabactin (*ybt*), colibactin (*clb*), aerobactin (*iuc*), 1 = *ybt* only, 2 = *ybt* and *clb* (or *clb* only), 3 = aerobactin (without *ybt* or *clb*), 4 = aerobactin with *ybt* (without *clb*), 5 = *ybt*, *clb* and *iuc*). Based on that we defined resistant cKp with a resistance score ≥1 and a virulence score <3 and convergent KP refers to isolates with at least one acquired ESBL gene and a virulence score ≥3 (Lam et al., 2021). Kleborate was primarily developed to screen the genome assemblies of species within the KpSC, including *K. pneumoniae*, *K. quasipneumoniae* and *K.* var*iicola*. The same thresholds are used to assign virulence and resistance scores, regardless of KpSC species. Therefore, we decided to apply the same criteria for classifying strains as “classical” (cKv), “hypervirulent” (hvKv) or “convergent” (conKv). Note that while convergent and hypervirulent KV isolates have been described, none have been used in this study (Lu et al., 2018).

**Table 1:** Overview of clinical *Klebsiella* spp. strains used in this study.

Species/ST	Strains included in omics	Original strain name	Source	Year of isolation	Pathotype	Reference
KV/NA	PBIO3543 (ST347)	275/15	Body fluid	2011	cKv	Becker et al. (2018)
	PBIO3544 (ST906)	328/15	Anal swab	2014	cKv	Becker et al. (2018)
KP/ST15	PBIO3546	331/15	Pleural fluid	2014	cKp	Becker et al. (2018)
	PBIO3547	334/15	Urine	2012	cKp	Becker et al. (2018)
KP/ST147	PBIO3553	313/15	Urine	2011	cKp	Becker et al. (2018)
	PBIO3554	315/15	Tracheal secretion	2012	cKp	Becker et al. (2018)
KP/ST258	PBIO3556	278/15	Urine	2012	cKp	Becker et al. (2018)
	PBIO3557	287/15	Urine	2014	cKp	Becker et al. (2018)
KP/ST307	PBIO3559	333/15	Central venous catheter	2014	cKp	Becker et al. (2018)
	PBIO1953	va20750	Tracheal secretion	2019	conKp	Heiden et al. (2020)

The sequence type (ST) of *K. pneumoniae* (KP) and *K. variicola* (KV) included in all phenotypic analyses are summarized. Information on the isolates including their source, year of isolation and original description is provided. The pathotype for KP isolates was manually assigned based on Kleborate resistance and virulence scores. cKp, classical KP; cKv, classical KV; conKp, convergent KP.

### 2.2 Cultivation and harvest of bacterial strains

KP together with *E. coli* are the most common bacteria in UTI which is a risk factor for development of severe infectious disease (Flores-Mireles et al., 2015; Paczosa and Mecsas, 2016; Oliveira et al., 2022). Therefore, all cultivations of bacterial strains were performed in synthetic human urine (SHU), composited according to Abbott et al. (2020) with shaking (150–220 rpm) at 37°C. For growth kinetics 5.0 mL of SHU were inoculated with a single colony of the respective strain (Table 1) and incubated overnight. Then, 10.0 mL of SHU were inoculated at an optical density of 0.05 at *λ* = 600 nm (OD_600_) and growth was monitored by measurements every 30 min for 8 h. In its original recipe SHU contains 6.8 mM citrate (Abbott et al., 2020), this was not added to the citrate depleted SHU to check for citrate-dependent growth phenotypes. Strains were grown overnight in either normal SHU or citrate depleted and from that cultures in a 24-well suspension plate (Sarstedt, Nümbrecht, Germany) were inoculated at OD_600 nm_ of 0.05 and incubated at 37°C with double orbital shaking (180 rpm) before each measurement every 30 min using a Spark® Multimode Microplate Reader (TECAN, Switzerland). To mitigate the variability in biological replicates, total protein and RNA were extracted from cultures that were initiated through a systematic serial dilution of 5.0 mL overnight cultures, originating from a glycerol stock. Subsequently, pre-cultures (20.0 mL of SHU) were inoculated at an OD_600_ of 0.05, starting from mid-exponential phase overnight cultures. Once the pre-cultures reached the mid-exponential phase again, the main cultures (100.0 mL of SHU) were inoculated in a similar manner. Cell harvest during the mid-exponential phase involved cooling in liquid nitrogen and centrifugation (3 min; 4°C; 8,000 x *g*). Cell pellets were stored at −80°C until RNA and protein preparation.

### 2.3 Isolation of chromosomal DNA and whole genome sequencing

Extraction of total DNA for all strains except PBIO1953 (which was previously sequenced; ERR4422314 (Heiden et al., 2020)) was performed using the MasterPure DNA Purification Kit for Blood, Version 2 (Lucigen, Middelton, WI, USA), according to the manufacturer’s instructions. Afterwards, DNA was quantified with dsDNA HS Assay Kit using Qubit 4 fluorometer (Thermo Fisher Scientific, Waltham, MA, USA) and then shipped to SeqCenter (Pittsburgh, PA, USA). Library preparation was performed using the Illumina DNA Prep Kit and IDT 10 bp UDI indices (Illumina, San Diego, CA, USA), hybrid whole genome sequencing was carried out on an Illumina NovaSeq X Plus (2 × 151 bp reads) and Oxford Nanopore R10.4.1 flowcells run on a MinION platform. Demultiplexing, quality control, and adapter trimming were performed using bcl-convert (v. 4.1.5) (Prjibelski et al., 2020).

### 2.4 Assembly and annotation

Raw sequencing Illumina reads were adapter- and quality-trimmed using Trim galore.^1^ The Trycycler pipeline (v. 0.5.3) (Wick et al., 2021) was used to generate most accurate hybrid assemblies by combining assemblies from flye (v. 2.9.2) (Kolmogorov et al., 2019), miniasm and minipolish (v. 0.1.3) (Wick and Holt, 2023) and raven (v. 1.8.1) (Vaser and Šikić, 2021). The combined assemblies were polished according to the pipeline instructions using Polypolish (v. 0.5.0) (Wick and Holt, 2022) and POLCA (from MaSuRCA v. 4.1.0) with the Illumina short-reads. Since Trycycler sometimes misses small plasmids, short-reads and long-reads were additionally assembled with Unicycler (v. 0.5.0) (Wick et al., 2017). Both assemblies were compared and missing plasmid contigs from Unicycler were added to the Trycycler output, thus we obtained a closed reference with all plasmids for each of the strains. For PBIO1953 the previously assembled closed genome (ERR4422314) was used (Heiden et al., 2020). All genomes were annotated with Bakta (v. 1.7.0, database version 5). All genomes were further analyzed with Kleborate (v. 2.3.2) (Lam et al., 2021) to verify the ST and determine resistance and virulence features.

### 2.5 Pangenome analysis

To establish the foundation for subsequent analyses, we curated a dataset that included the genomes of two different KV strains (PBIO3543 and PBIO3544) along with eight KP strains (PBIO3546, PBIO3547, PBIO3553, PBIO3554, PBIO3556, PBIO3557, PBIO3559, and PBIO1953) (Table 1). Utilizing the obtained closed genomes of these strains, we constructed a pangenome. Using the pangenome analysis pipeline Proteinortho (v. 6.2.3) (Lechner et al., 2011), we performed orthologous protein sequence clustering with coverage of ≥50.0% and e-value 1e-5 (other settings: default). The resulting pangenome was further subdivided into a core genome, containing genes shared by all strains included in the study, and an accessory genome, consisting of genes unique to specific subsets of strains included in the study (Tettelin et al., 2008; Rouli et al., 2015). The accessory genome was subdivided into the shell genome (genes present in a minimum of two and a maximum of nine strains), the cloud genome (genes unique to a single genome within the dataset), and to a group comprising genes shared by all KP strains but absent in KV strains, further referred to as markers. The visualization of the distribution of genes within the pangenome was performed using the ggplot2 package and RStudio (v. 4.3.1). A reference-independent, alignment-free method based on whole genome data was selected to compare the isolates of the two different species KP and KV using mashtree (v.1.2.0). To visualize the relationship of the used strains, we determined the genetic distance with mashtree (v.1.2.0), and plotted the resulting tree with iTOL (v.6.8.1) (Den Bakker et al., 2022) including metadata like ST, resistance score based on Kleborate (Lam et al., 2021), source of isolation, carriage of ESBL and carbapenemase genes, and selected marker genes. Notably, this pangenome dataset served as the database for subsequent transcriptomic and proteomic analyses.

### 2.6 Isolation of total RNA and sequencing

Total bacterial RNA was isolated by mechanical disruption and acid phenol-chloroform extraction as previously described with minor modifications (Nicolas et al., 2012) from mid-exponential growth phase. The bacterial pellet (equivalent to 8 OD units) was resuspended in 200 μL of ice-cold killing buffer (composition, 20.0 mM Tris–HCl, pH 7.5; 5.0 mM MgCl_2_; 20.0 mM NaN_3_). Subsequently, mechanical disruption was carried out using a Mikro-Dismembrator S (Sartorius, Goettingen, Germany) for 2 min at 2600 rpm. The resulting cell powder was resuspended in 2.0 mL of lysis buffer (composition, 4 M guanidine-thiocyanate; 25.0 mM sodium acetate, pH 5.2; 0.5% (wt/vol) sodium *N*-lauroylsarcosine) prewarmed at 50°C and then stored at −80°C until RNA preparation. The RNA extraction consisted of two washes with 1 volume of acid phenol solution (ROTI®Aqua-P/C/I, Carl Roth, Karlsruhe, Germany) and one round with 1 volume 24:1 chloroform-isoamyl alcohol (chloroform ROTIPURAN® ≥ 99%/IAA pH 8.0, Carl Roth). The upper phase was transferred into a fresh tube to avoid carry-over of DNA-containing interphase. Following the addition of 1/10 volume of 3.0 M sodium acetate, RNA was precipitated with 1.0 mL of isopropanol overnight at −20°C. Upon centrifugation (15 min, 15,000 rpm, 4°C) the resulting RNA pellet was washed twice with 70.0% (vol/vol) ethanol before the dried pellet was solubilized in 100 μL of RNase-free water. Samples were treated with rDNase (Macherey-Nagel, Düren, Germany) according to the manufacturer’s protocol to minimize DNA contamination. Purification was carried out using the RNA Clean-Up Kit (Macherey-Nagel). Finally, the RNA concentration was determined using a NanoDrop spectrometer (NanoDrop 8000, PeqLab, Erlangen, Germany).

RNA samples were shipped frozen to Novogene (Cambridge, United Kingdom) and, following rRNA depletion and mRNA library preparation, sequenced using 2 × 151 bp reads (Illumina NovaSeq 6000, stranded).

### 2.7 Differential gene expression

Trim Galore (v. 0.6.8)^2^ was used for the adapter and quality trimming of the raw sequencing reads. The trimmed reads were then mapped with Bowtie 2 (v. 2.5.1/mode: –very-sensitive-local) (Langmead and Salzberg, 2012), using the assemblies of the previously generated genomes as references for the individual strains (Table 1). The gene counts were calculated using featureCounts (v. 2.0.1/stranded-mode) (Liao et al., 2014) based on the annotation for the respective strain. Next, the gene names in the count tables were replaced with the cluster IDs of the pangenome and the different count tables were merged together. The modified count table was imported into RStudio (v. 4.3.1), and genes differentially expressed in comparisons between each KV strain and each KP strain, as well as among the KV strains themselves, were identified with DESeq2 (v. 1.40.0) in default mode. The obtained data were submitted to further analysis for identification of unique KP characteristics across omics levels (refer 2.12).

### 2.8 Isolation of intracellular proteins

Prior to isolation of intracellular proteins, samples from mid-exponential growth phase were washed twice with ice-cold phosphate-buffered saline (PBS; Thermo Fisher Scientific), and the pellet was suspended in 100 μL of a solution comprising of 20.0 mM HEPES (pH 8.0) and 2.0% (wt/vol) sodium dodecyl sulfate (SDS), followed by denaturation at 95°C for 1 min with vigorous shaking. Subsequently, the cells were disrupted using a Mikro-Dismembrator S (Sartorius, Göttingen, Germany) for 3 min at 2600 rpm. The obtained cell powder was then resuspended in 150 μL of preheated (95°C) 20.0 mM HEPES (pH 8.0) and transferred into a 1.5 mL low binding pre-lubricated tube (Sorenson™ BioScience Inc., Salt Lake City, UT). After cooling to room temperature (RT), 4.0 mM MgCl_2_ and 0.005 U/μL benzonase (Pierce Universal Nuclease, Thermo Fisher Scientific) were added to the lysates. Ultrasonication was performed for 5 min and the resulting cell debris was pelleted by centrifugation (30 min; RT; 17,000 x *g*). The resulting supernatants containing soluble protein lysates were transferred into a fresh 1.5 mL low binding pre-lubricated tube (Sorenson™ BioScience Inc.) to ensure optimal conditions for further downstream analysis.

### 2.9 Bicinchoninic acid assay

In order to standardize protein amount for subsequent mass spectrometric analysis, protein concentration was quantified as previously described (Blankenburg et al., 2019; Reder et al., 2023) using the MicroBCA™Protein Assay Kit (Thermo Fisher Scientific) according to the manufacturer’s instructions. Briefly, samples were diluted at a 1:50 ratio. Concurrently, standards containing a known concentration of bovine serum albumin were prepared to match the concentrations of SDS and HEPES in both the samples and standards. All samples and standards were each mixed with equal volumes of the working reaction in duplicates and incubated at 68°C for 1 h. The standards were measured first, followed by sample measurements, all performed in duplicates using the OT-2 robot (Opentrons, Long Island City, NY) and BioTek Synergy (Agilent Technologies, Waldbronn, Germany). The acquired data were evaluated using the MassSpecPreppy Shiny application (v. 1.1.1) based on the determined standard curves for accurate quantification (Reder et al., 2023).

### 2.10 Single pot solid-phase enhanced sample preparation (SP3)

Prior mass spectrometric analysis the samples were prepared using the Single pot solid-phase enhanced sample preparation (SP3) protocol as described by Blankenburg et al. with slight modifications (Blankenburg et al., 2019). For the trypsin digestion, 5 μg of total protein in 10 μL of 20.0 mM HEPES (pH 8.0) were incubated (18 min shaking at 1400 rpm) with 10 μL of equal volumes of hydrophilic (Speedbead magnetic carboxylated modified particles, GE Healthcare, United Kingdom) and hydrophobic (Sera-Mag Speedbead carboxylated-modified particles, Thermo Fisher Scientific) magnetic beads. The supernatant was discarded by sedimentation of beads on a magnetic rack, followed by two washing steps with 70.0% (vol/vol) ethanol and one washing step with 100% (vol/vol) acetonitrile (ACN) before air-drying. Trypsin digestion was performed in freshly prepared 20.0 mM ammonium bicarbonate buffer at trypsin to protein ratio of 1:25 for 18 h at 37°C. The digestion process was stopped by the addition of ACN to a final concentration of 95.0% (vol/vol) (18 min shaking at 1400 rpm). The beads were further washed and desalted with 100% (vol/vol) ACN and air-dried before elution in 10 μL of 2.0% (vol/vol) DMSO by sonication for 5 min. The beads were then pelleted by pulse centrifugation, and the resulting supernatant was transferred to a fresh microcentrifuge tube. The eluate was subsequently diluted with an equal volume of buffer A containing 2.0% (vol/vol) ACN and 0.2% (vol/vol) acetic acid and stored at −20°C until mass spectrometry.

### 2.11 Acquisition and analysis of mass spectrometry data

Peptides were separated using an UltiMate 3,000 nanoLC device (Thermo Fisher Scientific) with a pre-column (Acclaim PepMap; Thermo Fisher Scientific) and an analytical column (Accucore; Thermo Fisher Scientific) by applying a binary gradient with buffer A [0.1% (vol/vol) acetic acid in HPLC-grade water] and buffer B (0.1% (vol/vol) acetic acid in ACN) at a flow rate of 300 nL/min. After ionization, peptides were analyzed with a Q ExactiveTMHF mass spectrometer (Thermo Fisher Scientific) in data independent acquisition (DIA) mode. Specification of the used gradient and detailed settings on nanoLC-MS/MS data acquisition are provided (Supplementary Table 1). Raw data were mapped against in-house established pangenome database including whole genome sequences of all analyzed strains using Spectronaut (v. 18.1) (Biognosys, Schlieren, Switzerland). All search parameters are provided (Supplementary Table 2). In brief, trypsin/P was set as the digesting enzyme with 0 missed cleavage allowed. Oxidation at methionine was set as variable modification. Peptides with min. Seven amino acids displaying a *q*-value cut-off for detection of <0.001 were selected for further analyses. Only proteins detected with at least two peptides were considered for statistical analysis, which was performed using RStudio (v. 4.1.3 (2022-03-10)) with the tidyverse package (v. 1.3.2). Normalization factors were obtained from Spectronaut analysis. Missing values (intensity = 0) were replaced with the half-minimal intensity value from the whole dataset. Detected methionine oxidized peptides were excluded from further quantitative analysis. Ion intensities per sample and peptide were summed to calculate peptide intensities. Protein intensities were calculated in the Spectronaut software as intensity-based absolute quantification (iBAQ) values. With the iBAQ algorithm, the summed intensities of the peptides of one protein are divided by the number of theoretically observable peptides (summarized in (Rozanova et al., 2021)). iBAQ intensities were separated into quantiles by regarding intensities in the lower two quantiles as close to detection limit. Statistical comparison was accomplished on peptide level with the PECA package (v. 1.30.0) by applying a ROTS test (ROPECA approach - reproducibility-optimized peptide change averaging) (Suomi and Elo, 2017). Proteome data have been stored at the ProteomeXchange Consortium via the PRIDE partner repository (Perez-Riverol et al., 2019) with the dataset identifier PXD047744.

### 2.12 Identification of unique KP characteristics across omics levels

The investigation of KP’s unique features was conducted at genomic, transcriptomic, and proteomic levels, and aimed to identify genes that are uniquely present or induced in SHU in all scrutinized KP strains compared to their KV counterparts, and to KQ on genomic level (Table 1; Supplementary Tables 3, 4). At the genomic level, we selected for genes by filtering for presence in all KP genome and absent in the KV genomes (Supplementary Table 3). The evaluation of transcriptomic and proteomic data based on the pangenome followed a systematic approach. Transcriptomic and proteomic profiles of each KP strain were compared individually with each KV strain. In addition, comparisons were made among the KV strains themselves. A gene was considered differentially expressed and a protein as differentially abundant if the absolute value of the log2 fold change (L2FC) was equal to or greater than 1.5, and the adjusted *p*-value was less than or equal to 0.05 (|L2FC| ≥ 1.5; p-value adjusted≤0.05). Subsequently, genes were filtered to include only those differentially expressed in all eight KP strains when compared to both KV strains, excluding those differentially expressed among the KV strains. The cumulative set of genes meeting these criteria was denoted as genomic, transcriptomic or proteomic markers (Supplementary Tables 3, 4), meaning characteristics, that are unique among the KP strains. The transcriptomic and proteomic markers encompass genes that may also be present in the genome of KV but are not expressed in SHU, as well as genes absent in KV genomes (differentially encoded), yet appearing differentially expressed in KP. Still these genes have been included since this approach aimed to understand the complex interplay of genomic differences and expression patterns on transcriptomic and proteomic levels. The different transcriptomic and proteomic profiles were visualized using the pheatmap package in RStudio (v. 4.3.1).

### 2.13 Homologous clustering-approach

To contextualize the identified genes in a broader genetic background, we downloaded all publicly available KP, KV, and KQ genomes from the National Center for Biotechnology Information (NCBI) database (Sayers et al., 2022) (25 Aug 2022). Kleborate was used to assign species, ST and resistance and virulence score to every genome. We filtered the genomes for eight globally recognized KP STs: ST11, ST15, ST23, ST101, ST147, ST258, ST307 and ST512, in total 5,758 KP genomes along with 982 genomes of KV and KQ (Supplementary Table 5; Supplementary Figure 1) (Wyres et al., 2020a). Prediction of protein coding genes of downloaded assemblies was performed using Prokka (v. 1.14.6) (Seemann, 2014). A time- and resource-saving approach was chosen to cluster all protein sequences from the KP, KV and KQ genomes using cd-hit (v. 4.8.1) (Fu et al., 2012). This dataset will be further referred to as homologous clustering. Very strict thresholds of 95.0% sequence identity and sequence coverage were applied to ensure that only very similar sequences were included within a cluster. For further classification, we borrowed the terminology for pangenome analyses, and classified the clusters analogous to the previous generated pangenome into a core (present in ≥95.0% of the genomes), a shell (present in ≥15.0, <95.0% of the genomes), a cloud (present in <15.0% of the genomes) and the group with the markers (present in ≥95.0% of the KP, <10% of the KV and KQ genomes). Note that distinct clustering cut-offs were chosen for the two approaches (pangenome and homologous clustering). This decision was guided by varying sample sizes, the consideration of syntenies during the construction of the pangenome (most assemblies of the clustering-approach were not closed), and the aim to detect variations in the sequences within the extensive homologous clustering. We then identified which gene from the pangenome analysis belonged to the respective clusters in the homologous clustering by using BLASTP (BALST+ v.2.13.0, −task blastp) and manual curation (80% identity and 80% sequence coverage).

### 2.14 Functional categorization

For in-depth analysis, genes matching the previously described criteria for the so-called markers underwent examination regarding their physiological functions concerning bacterial fitness and virulence. This assessment incorporated Bakta and eggNOG online (v. 2.1.9) annotations (Huerta-Cepas et al., 2019; Cantalapiedra et al., 2021). The Kyoto Encyclopedia of Genes and Genomes (KEGG) (Kanehisa et al., 2023) from eggNOG annotation was used for dissecting the involvement of specific genes within their respective functional pathways. Information about the cluster of orthologous groups (COG) was plotted for genes up- or down-regulated on transcriptomic or proteomic levels. However, COG annotation could not be determined for all genes (Supplementary Figure 2). To compensate for this limitation, our functional analysis was expanded through the creation of specialized functional categories (Supplementary Tables 6, 7). These categories were crafted to include the distinctive characteristics of all markers and summarize them following a similar structure of the COG database. The determination of these characteristics drew from information sourced from the KEGG and associated databases (including UniProt and STRING), ensuring that each gene was systematically classified into only one of the categories (Snel et al., 2000; Szklarczyk et al., 2021; Consortium T.U, 2023; Kanehisa et al., 2023). This approach not only provided a holistic understanding of the functional landscape but also facilitated the identification of unique features among KP. Upon categorization, we went beyond to closely study how selected markers reveal a potential association with to bacterial virulence and fitness by incorporating previously published literature.

### 2.15 Software and statistical analysis

All phenotypic experiments were performed with three or more independent biological replicates and respective statistical analyses were performed using GraphPad Prism (v. 9.3.0) for Windows (GraphPad Software, San Diego, CA, USA). Data were expressed as mean and standard deviation. Assessment of statistical significance was performed via one-way analysis of variation (ANOVA) with uncorrected Fisher’s Least Significant Difference or unpaired parametric t-test using Bonferroni-Dunn correction for multiple comparisons. Values lower than 0.05 were used to show significance (*p < 0.050; **p < 0.010; ***p < 0.001). For transcriptomic and proteomic analyses, a |L2FC| > 1.5 with an adjusted *p*-value equal or smaller than 0.050 was considered as significant. We used large language models, such as ChatGPT (GPT-4), to assist in revising portions of the manuscript. All AI-generated content was thoroughly reviewed, edited, and approved by the authors to ensure its accuracy and integrity.

## 3 Results

### 3.1 Pangenome analysis revealed differences among KP and KV strains, but also similarities

To uncover distinctive traits of KP compared to other species within the KpSC, we curated a set of ten KP and KV strains from our collection for multi-omics analysis. This set comprised two strains each of four highly successful, international high-risk KP STs recognized for their MDR and/or virulence profiles according to Kleborate (Lam et al., 2021), alongside two distinct STs of KV, all of them carbapenemases- and or ESBL-producers (Becker et al., 2018; Heiden et al., 2020).

**Figure 1:**
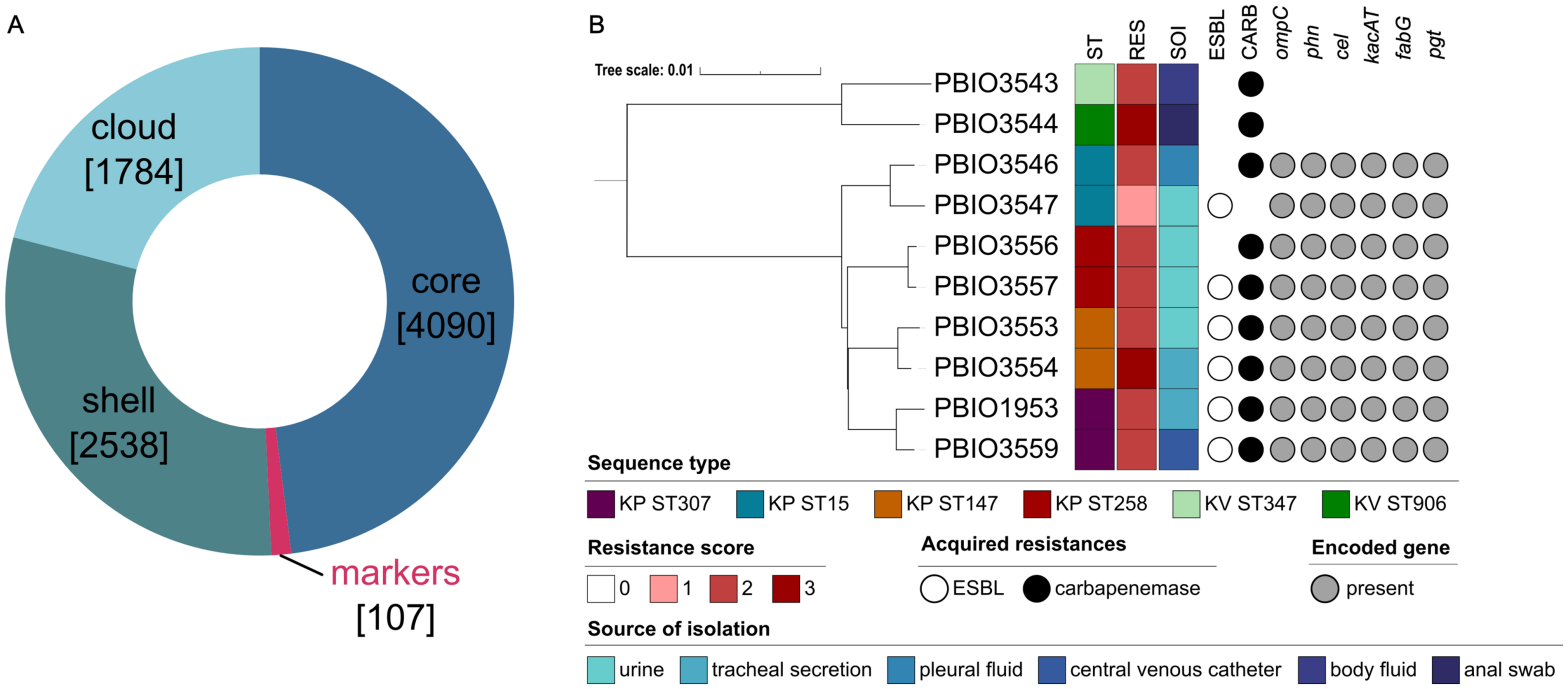
Pangenome analysis and genetic distance-based tree (mashtree) of the selected strains. **(A)** Pangenome analysis grouped genes regarding their occurrence in all strains (core), in minimum two and maximum nine strains (shell), in only one strain (cloud) or in all eight *K. pneumoniae* (KP) but not the *K. variicola* (KV) strains (markers). Numbers in square brackets indicate the number of genes within the respective category. **(B)** Based on the whole genomic data, the analyzed dataset including eight different KP and two KV strains was analyzed regarding its phylogenetic relationships considering relevant metadata like the sequence type (ST), resistance score (RES), acquired resistances (extended-spectrum *β*-lactamase [ESBL], carbapenemases [CARB]) and the source of isolation (SOI). The resistance score was determined based on Kleborate (0 = no ESBL, no CARB (regardless of colistin resistance); 1 = ESBL, no CARB (regardless of colistin resistance); 2 = CARB without colistin resistance (regardless of ESBL genes or OmpK mutations); 3 = CARB with colistin resistance (regardless of ESBL genes or OmpK mutations)). Additionally, the presence or absence of selected groups of marker genes based on KEGG analysis is shown. The phylogenetic tree was constructed using iTOL (v. 6.8.1) and edited with Inkscape.

Our aim was to examine a comprehensive range of genetic, transcriptomic, and proteomic factors. First, we investigated the distribution of genes among all ten genomes by performing a pangenome analysis using Proteinortho (Figure 1A). This revealed a total number of 8,519 genes within the pangenome, from which 48% were shared by all strains (core genome). The shell genome consists of genes shared by at least two and a maximum of nine strains and accounts for 30% of the gene content. Notably, around 10% of the shell genome (*n* = 256 genes) comprises genes unique to KV (Supplementary Table 3). However, further analysis of these unique genes was not conducted, as it was beyond the scope of this study. The cloud genome included 1,784 genes, which were uniquely present in only one strain (approximately 21.0%). Finally, the most interesting result was a group we refer to as “genomic markers from the pangenome” (GM) including 107 genes (1.3%) present in all KP but absent in the KV strains (Supplementary Table 3). According to the KEGG database, the GM were associated with diverse functions in metabolic pathways, encompassing carbohydrates, lipids, nucleotides, amino acids, and other secondary metabolites. We found an enrichment of gene functions related to membrane transport mechanisms, such as ABC transporters, the phosphotransferase system (PTS), and two-component systems. Among others, respective genes encoded proteins of the cellobiose PTS (*cel* cluster), ensuring uptake and utilization of this carbohydrate. Another example was *fabG*, which is involved in fatty acid metabolism. As stated above, genes with functions in amino acid, but also nucleotide metabolism, were quite prominent among the GM including the *phn* gene cluster encoding ABC transporter components enabling the sensing and utilization of phosphonates. In contrast to the KV strains, KP also encoded a *pgt* gene cluster of the two-component system responsible for phosphoglycerate transport. In addition to these metabolism-related genes, we found *ompC* encoding an outer membrane porin and *kacAT* encoding a type II toxin-antitoxin as unique genes of the KP strains among the GM (Figure 1B).

Mashtree in combination with Kleborate analysis was performed to understand the dataset in more detail. As expected, the mashtree analysis showed a clear separation of the KV from the KP genomes, with the latter closely clustering together, including the respective STs. More importantly, Kleborate revealed a resistance score of >2 for all except one strain (PBIO3547). In addition to *ampC,* each strain carried a minimum of one additional *β*-lactamase gene. In five of eight KP strains, genes encoding for both ESBL and carbapenemases were detected. Note that all strains originate from human sources with KV strains obtained from body fluid and an anal swab (PBIO3543 and PBIO3544, respectively) (Becker et al., 2018). Four KP strains were isolated from urine (PBIO3547, PBIO3553, PBIO3556, and PBIO3557), two from tracheal secretion (PBIO3554, and PBIO1953), and one from pleural fluid (PBIO3559) (Becker et al., 2018; Heiden et al., 2020).

In summary, pangenome analysis of the dataset demonstrated differences between the two species, impacted by the presence of KP-specific genes associated with metabolic features.

### 3.2 Transcriptome and proteome analysis in urine-like conditions demonstrated similar regulatory patterns among KP in contrast to KV strains

**Figure 2:**
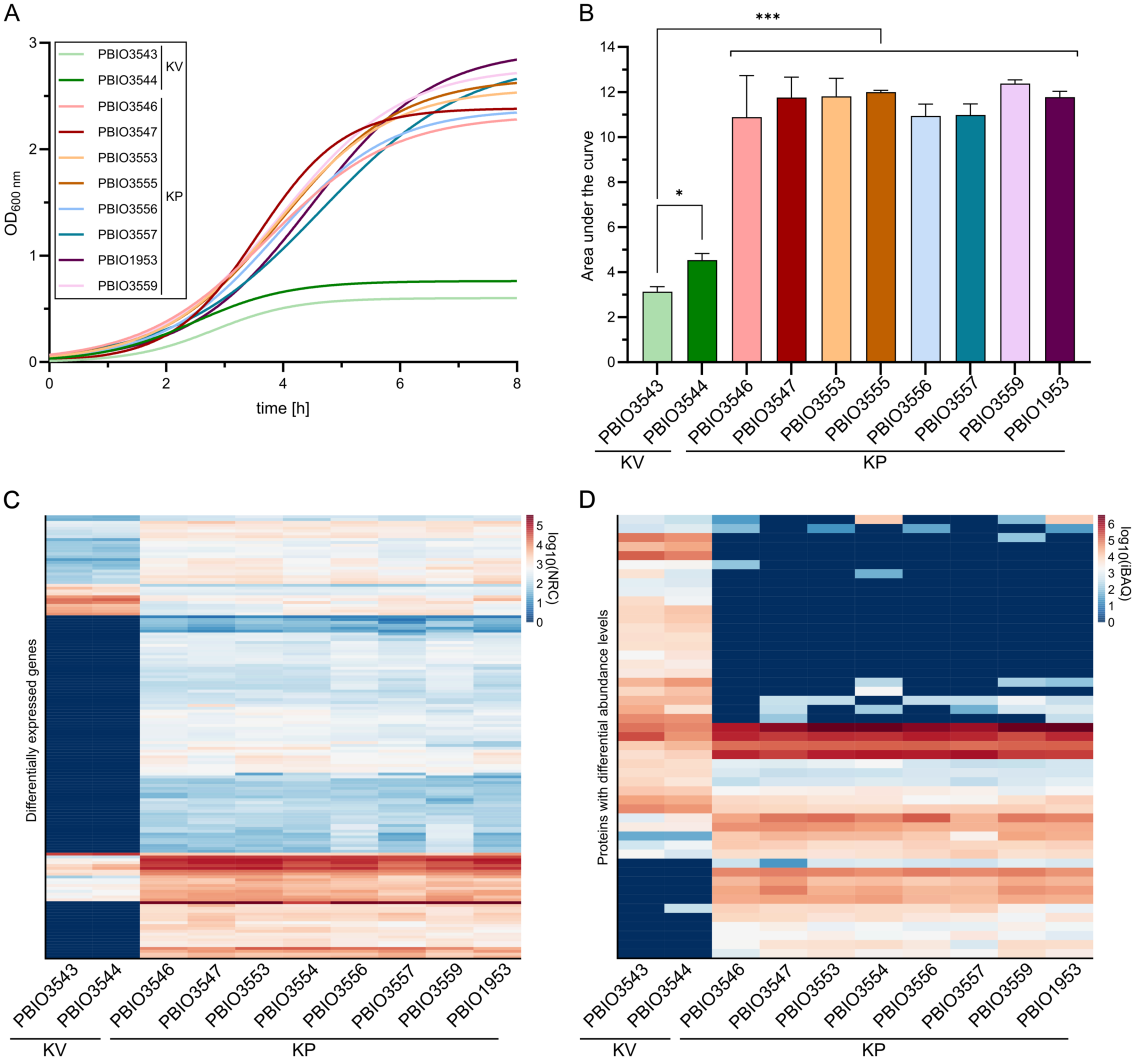
Growth kinetics and expression profiles of *K. pneumoniae* (KP) and *K. variicola* (KV) strains in SHU. Strains were cultivated in SHU in biological triplicates. **(A)** Growth kinetics are displayed using GraphPad Prism and Gompertz-Growth fitting. **(B)** The area under the curve was calculated from three individual cultivations (*n* = 3). The significance of differences was calculated using one-way ANOVA (*p < 0.050; **p < 0.010; ***p < 0.001). **(C + D)** The normalized read counts (NRC) obtained from transcriptomic analysis **(C)** and the intensity-based absolute quantification (iBAQ) obtained from proteomic analysis **(D)** are plotted for each strain. The regulatory profiles of transcriptomic markers (TM) and proteomic markers (PM) are shown as separate heatmaps. The decimal logarithm (log_10_) was applied to all values for better visualization of differences.

Following the genomic analysis, which revealed 107 GM in KP, we next screened for differentially expressed genes and differentially abundant proteins to (i) explore whether the GM would translate to the transcriptome and/or proteome, and (ii) to identify different core markers and regulatory patterns on transcriptomic and/or proteomic levels. Given that KP is a leading cause of UTI, we sampled in SHU, which likely reflects relevant nutritional conditions of urine (Flores-Mireles et al., 2015; Paczosa and Mecsas, 2016; Oliveira et al., 2022). Interestingly, KP grew significantly better in SHU than the KV strains (*p* < 0.001) (Figures 2A,B). However, this distinct difference between KV and KP strains was not observed when grown in LB medium and only one of the KV strains showed slightly diminished growth (Supplementary Figures 3, 4). Combined with the genomic data presented, that revealed KP-specific genes for phosphonate (*phn*) phosphoglycerate (*pgt*) and cellobiose (*cel*) transport (Figure 1B), this indicates a superior capacity for nutrient utilization by the KP compared to KV strains. Such enhanced nutrient utilization is particularly crucial in environments with limited nutrient availability, such as the urinary tract (Ipe and Ulett, 2016; Abbott et al., 2020). Note that this not only applied to the strains obtained from UTI but all KP isolates that were used in this analysis and isolated from different sources. Transcriptomic and proteomic analyses for all eight KP and two KV strains were performed to explore the underlying processes potentially supporting enhanced growth of KP and revealed major differences between KP and KV at both levels within the principal component analysis. In addition, differences among the KP were smaller and strains of the same ST clustered closely together (Supplementary Figure 5). Next, we compared the data of each KP with each KV strain and the two KV strains with each other. Genes that appeared significantly expressed (|L2FC| ≥ 1.5; *p*-value adjusted≤0.05) in all comparisons of KP vs. KV strains, but not between the two KV strains, were referred to as transcriptomic markers (TM) and proteomic markers (PM), respectively. By plotting the decimal logarithm (log_10_) of the normalized read counts (NRC) and the log_10_ of iBAQ values of all genes fitting the previously mentioned criteria, we obtained the regulatory patterns of all TM and PM. Comparison of these markers revealed that the overall patterns were seemingly different on transcriptomic versus proteomic levels (Figures 2C,D). However, they clearly show that all KP strains, independent of their ST, shared a similar expression profile, which was different from the two KV strains.

In general, the number of genes detected in the TM (*n* = 155) group was higher than in the PM (*n* = 48) group (Supplementary Figure 6). Moreover, a higher number of genes was upregulated in all individual comparisons of KP vs. KV on transcriptomic levels. Upregulated genes within the TM included 143, while only a total number of 12 genes were downregulated. Detected up- and down-regulations on proteomic levels were rather equal for the individual comparisons, with a slightly higher number of downregulations (*n* = 28) than upregulations (*n* = 20) among the PM. The total number of regulated genes suggests that the strains responded highly individually to the specific growth conditions in SHU. Nevertheless, a substantial overlap in markers across the different KP strains within each omics layer (155 TM, 48 PM) indicates common response mechanisms in KP that were absent in KV. Please note, that TMs and PMs must be classified into (i) genes that occur exclusively in KP (GM) or KV and are therefore considered as differentially expressed, and (ii) genes that occur in both KV and KP (core genome). Among the 155 TM genes, 103 were also detected as GM, while 52 were found in the core genome of the selected dataset. Of the 49 genes belonging to the PM, six were discovered as GM, 24 were assigned to the core, and 19 to the shell genome of the selected dataset. A total of 11 genes were classified as both TM and PM, 5 of which are also encoded in KV genomes (core genome) and 6 of which are only found in KP genomes (GM). This multi-omics analysis highlights that more than 96.0% of GM exhibited transcriptional activity, with detectable RNA levels under conditions mimicking urine. However, it also suggests that only a minority undergoes sufficient translation to be detected by proteomics under these experimental conditions. In addition, we identified genes from the core genome present in all strains of the dataset that exhibited unique expression patterns in KP strains, thereby being integral components of the groups of TM and/or PM. This suggests that the growth advantages observed in KP strains within SHU may not solely stem from their specific genetic makeup. Instead, they may be bolstered by shared regulatory and response mechanisms, which collectively enhance their overall fitness in nutrient-limited environments like the urinary tract.

### 3.3 In-depth characterization revealed common functional pathways on transcriptomic and proteomic levels in KP

**Figure 3:**
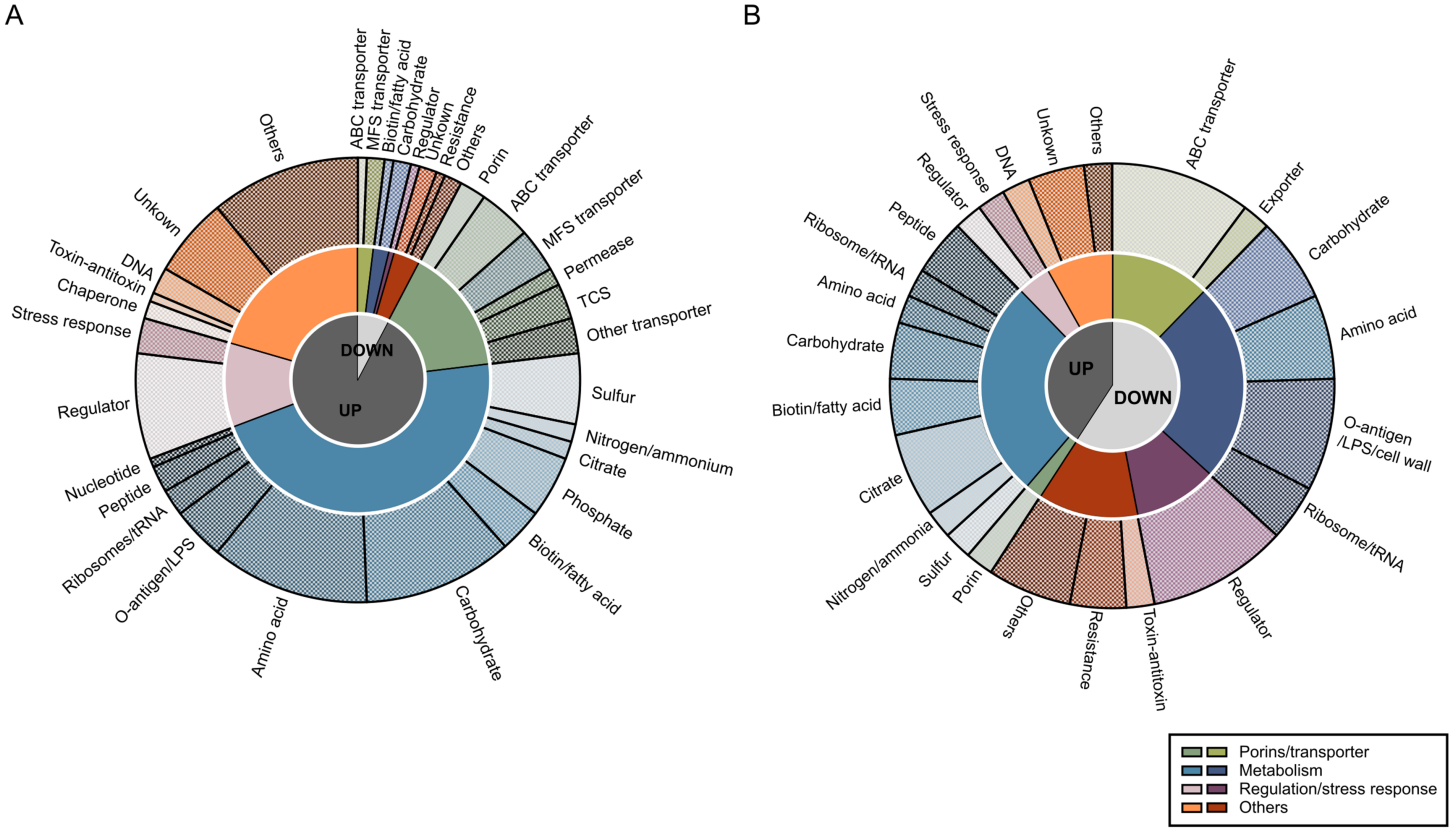
Functional analysis of transcriptomic markers (TM) and proteomic markers (PM). Functional evaluation of all TM **(A)** and PM **(B)** was performed by creating categories on three different levels of specificity based on all regulations. First, we only considered log2 fold changes (L2FC) > 1.5 (up-regulated [UP]) or < −1.5 (down-regulated [DOWN]). Second, four different groups were assigned for broad categorization: porins/transporter, metabolism, regulation/stress response, and others, as indicated in the legend. Third, more specific categories within the previously differentiated groups were chosen and each of the TM and PM was assigned to these categories. Visualization was done using GraphPad Prism and Inkscape.

The observed regulatory profiles on transcriptomic and proteomics levels for KP were further investigated regarding their physiological functions. Since categorization based on the COG database was insufficient (Supplementary Figure 2), specialized categories were manually assigned using information from eggNOG (e.g., COG, KEGG), BLASTP and literature research (Supplementary Table 6). Genes were first differentiated based on their up- or downregulation and abundance of proteins. Then, they were clustered into four different groups, namely porin/transporter, metabolism, regulation/stress response, and others (Figure 3). To facilitate a detailed characterization, more specific categories were assigned to all regulated genes (Supplementary Table 7). Indeed, transcriptomic and proteomic profiles not only varied in their number of up- or down-regulated genes and abundance of proteins but also in their putative functions. Nevertheless, some similarities were found. For instance, regulation of metabolic pathways was seemingly a key mechanism when growing KP in SHU. On transcriptomic levels, 49.0% of the up- and 25.0% of the down-regulated genes were assigned to this group, while on proteomic levels, 65.0% of proteins with higher abundance in KP compared to KV and 41.0% of proteins with lower abundance in KP compared to KV were involved. Moreover, most of the more specific categories were shared on both transcriptomic and proteomic levels, namely sulfur, nitrogen/ammonia, phosphate, biotin/fatty acid, carbohydrate, amino acid, ribosomes/tRNA, peptide, and citrate metabolism. Downregulations or lower abundance also affected transporters, with 25.0% on transcriptomic and 21.0% on proteomic levels. Genes with regulatory functions and involved in stress response had a greater impact on the proteomic (10.0% up- and 17.0% downregulated, respectively) than on the transcriptomic level (12.5% up- and 8.0% downregulated, respectively). Downregulation of genes and lower abundance of proteins involved in resistance, e.g., penicillin or chromate resistance, was observed, while genes for DNA replication and repair were upregulated and showed a higher abundance of corresponding proteins. Regulations and protein abundances also varied concerning toxin-antitoxin systems and chaperones. Overall, the in-depth characterization revealed that the regulatory patterns on a functional level are rather similar on transcriptomic and proteomic levels and that they mainly concern metabolic pathways.

### 3.4 Comparing regulated genes to a large set of KpSC genomes revealed unique markers and regulatory mechanisms in KP

**Figure 4:**
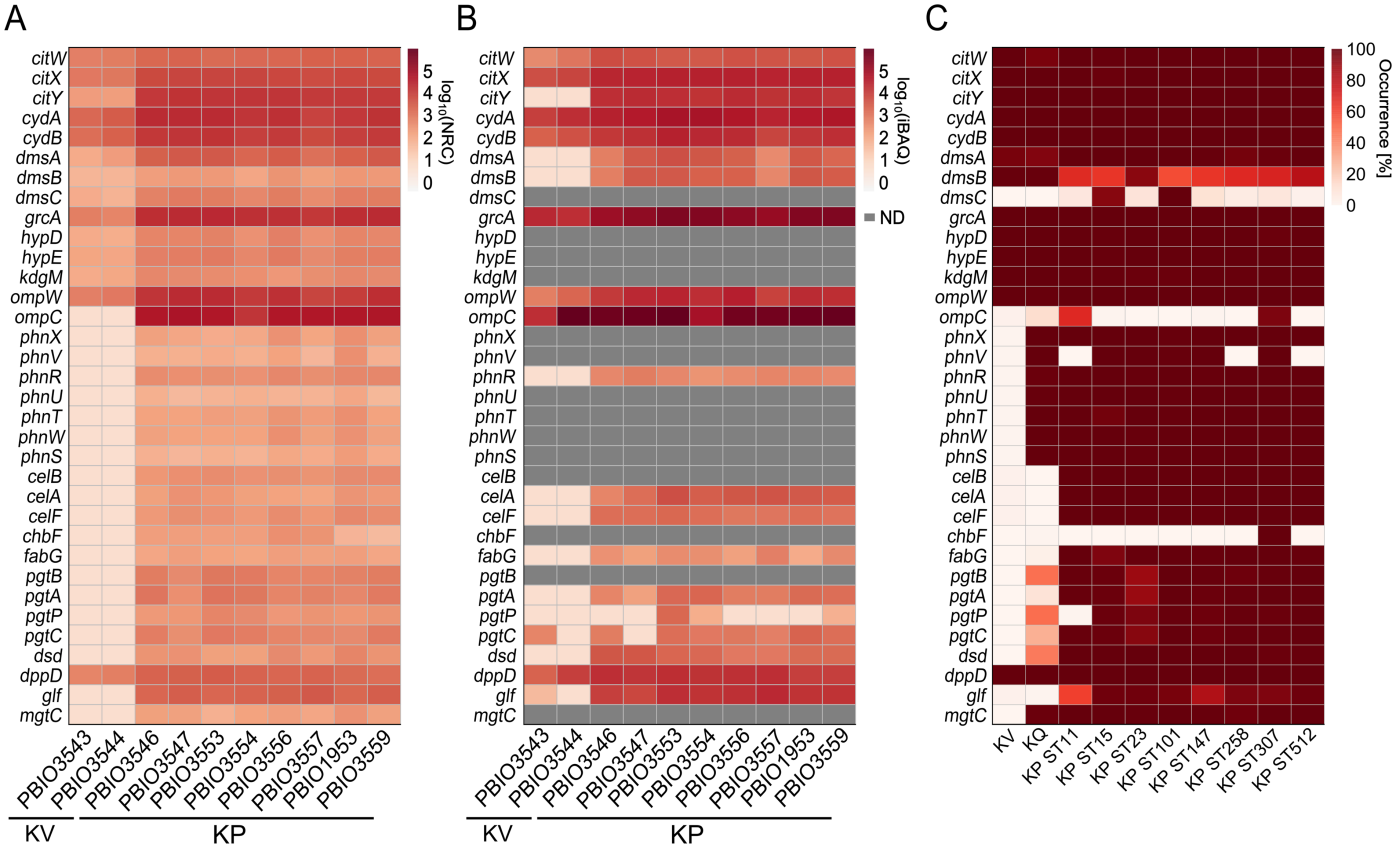
Detection of corresponding gene expression on multi-omics levels. Genes identified as differentially expressed on transcriptomic and/or proteomic levels were selected regarding their biological relevance based on literature research, as described. Heatmaps for all profiles, either expression on transcriptomic or proteomic level or occurrence on genomic level are shown and respective genes, strain or sequence type (ST) are indicated. ND means the protein was not detectable. **(A)** Transcriptomic profile of selected genes was generated by mapping the decimal logarithm (log_10_) of the normalized read counts (NRC) for each analyzed strain. **(B)** Profile of the expression detected on proteomic level of selected genes was generated by mapping the log_10_ of the intensity-based absolute quantification (iBAQ) values for each analyzed strain. **(C)** Selected genes were mapped against the homologs clustering (BLASTP).

The 193 differentially expressed genes on transcriptomic and/or proteomic levels between KP and KV in SHU were then further investigated to explore whether they would play a role as markers of the investigated KP STs in a broader context. We therefore leveraged 6,740 publicly available genome assemblies comprising KV and KQ as less prevalent representatives and eight different, international high-risk KP STs (i.e., ST11, ST15, ST23, ST101, ST147, ST258, ST307, and ST512) (Supplementary Figure 7) for this clustering-approach (Supplementary Table 5). All proteins were clustered based on a sequence identity and coverage greater than and equal to 95.0%, to also decipher species-conserved variations within the protein sequence. This resulted in 192,172 clusters within the cloud genome, 2,785 within the shell genome, and 3,083 clusters within the core genome. Additionally, we conducted an investigation to identify clusters primarily associated with KP genes (maximum of 10.0% of each of the KV and KQ genomes and a minimum of 95.0% of each KP ST represented within the cluster) and identified 273 genomic markers from homologous clustering (GM-L). By comparing the TMs and PMs with the clustering approach, we identified genes that were (i) included in the GM-L and differentially expressed in the transcriptome and/or proteome (*n* = 31), or (ii) differentially expressed (either on transcriptomic and/or proteomic levels) but which did not match the criteria for being included in the GM-L (*n* = 162) (Supplementary Figure 8). This demonstrates that genes, which were present in all genomes of the dataset, showed unique induction in KP in urine-like conditions. As mentioned above, most of the regulations addressed metabolic mechanisms (Figure 3). An in-depth literature and database (including KEGG, UniProt and STRING) review allowed us to filter for relevant regulations within the different categories of markers. To address the knowledge gap of explaining KP’s clinical significance, this analysis focused on those potentially exhibiting meaningful infection-biological relevance (Table 2) (Snel et al., 2000; Szklarczyk et al., 2021; Consortium T.U, 2023; Kanehisa et al., 2023). Form this filtering, the most interesting example for the first category (i), includes genes for a cellobiose PTS (*celABF*) that were upregulated on transcriptomic and/or proteomic levels in urine-like conditions in KP and absent in KV/KQ while present in the majority (≥95.0%) of all KP genomes (Figure 4). It is well-known that bacteria encode several variants of cellobiose-specific uptake systems (Schaefler and Malamy, 1969). Hence, we investigated the presence of gene variants of *celB* in a representative strain (PBIO1953, ST307). This showed that further genes with similarity to *celB* occurred to a maximum of 30.0% sequence identity in the ST307 isolate PBIO1953. Another interesting candidate of this category was *fabG*, which has a central function in fatty acid biosynthesis. A group of genes found in the second category (ii) which was present in >99.8% of the investigated genomes, encodes for a cytochrome ubiquinol oxidase (*cydAB*), coupling the reduction of molecular oxygen to the generation of a proton motive force and thus energy production. In addition, on transcriptomic levels, we found two gene clusters upregulated in KP strains, which are responsible for the acquisition of different phosphorus sources. The first cluster included seven genes of a phosphonate ABC-transporter (*phnRXVTWSU*). While these genes were absent in the majority of KV genomes, they were present in most KQ and KP genomes. A similar pattern was observed for the second gene cluster encoding genes for a two-component system involved in phosphoglycerate transport (*pgtABCP*). This gene cluster did not occur in KV but in 10.0–50.0% of the KQ genomes and in more than 92.0% of the KP genomes. In addition, genes involved in citrate uptake were identified in the second category (ii). While they occurred equally in all genomes of the large clustering approach, they were only induced in KP suggesting unique regulatory mechanisms (Figure 4).

**Table 2:** Selected differentially expressed genes with particular biological relevance.

Gene names	Description	Occurrence (category)	Previous observations	Source
*citWXY*	Citrate uptake	(ii)	Non-essential, but facilitates citrate utilization, two-component system requires careful regulation	Reinelt et al. (2003) and Chen et al. (2009)
*cydAB*	Cytochrome ubiquinol oxidase	(ii)	Generates a proton motive force through reduction of molecular oxygen promotes bacterial virulence, resistance and evasion of the host immune system	Giuffrè et al. (2014)
*phnRXVTWSU*	Phosphonate ABC-transporter	(ii)	Provides ability to utilize alternative phosphorus sources	Ivan et al. (2014) and Poehlein et al. (2017)
*pgtABCP*	Phosphoglycerate transport	(ii)	Two component system and transport detected as virulence factor in ExPEC strains	Kong et al. (2017)
*celABF*	Cellobiose PTS	(i)	Enables cellobiose uptake, which is not digested by humans *celB* mutants show reduced virulence and biofilm formation	Wu et al. (2012)
*fabG*	3-Oxoacyl-ACP reductase	(i)	Enables lipid supply for production and maintenance of the cell envelope	Ramos et al. (2018)

The gene names, a description, as well as summary of previous observations and the respective sources are shown for selected groups of genes which resulted from the in-depth multi-omics analysis. The groups are (i) included in the genomic markers from homologous clustering (GM-L) and differentially expressed in the transcriptome and/or proteome, and (ii) differentially expressed (either on transcriptomic and/or proteomic levels) but did not match the criteria for being included in the GM-L.

Integrating the smaller, deeply characterized dataset with the extensive genomic dataset enhanced resolution and robustness of the analysis. By including a higher diversity of genomes and incorporating genomes of another species (KQ) into the clustering approach, this strategy improved the categorization of marker. Moreover, it enabled a more nuanced interpretation of their distribution across a broader range of KP genomes and sequence types.

### 3.5 Genetic and regulatory diversity in citrate pathways influences adaptation to urine-like conditions

**Figure 5:**
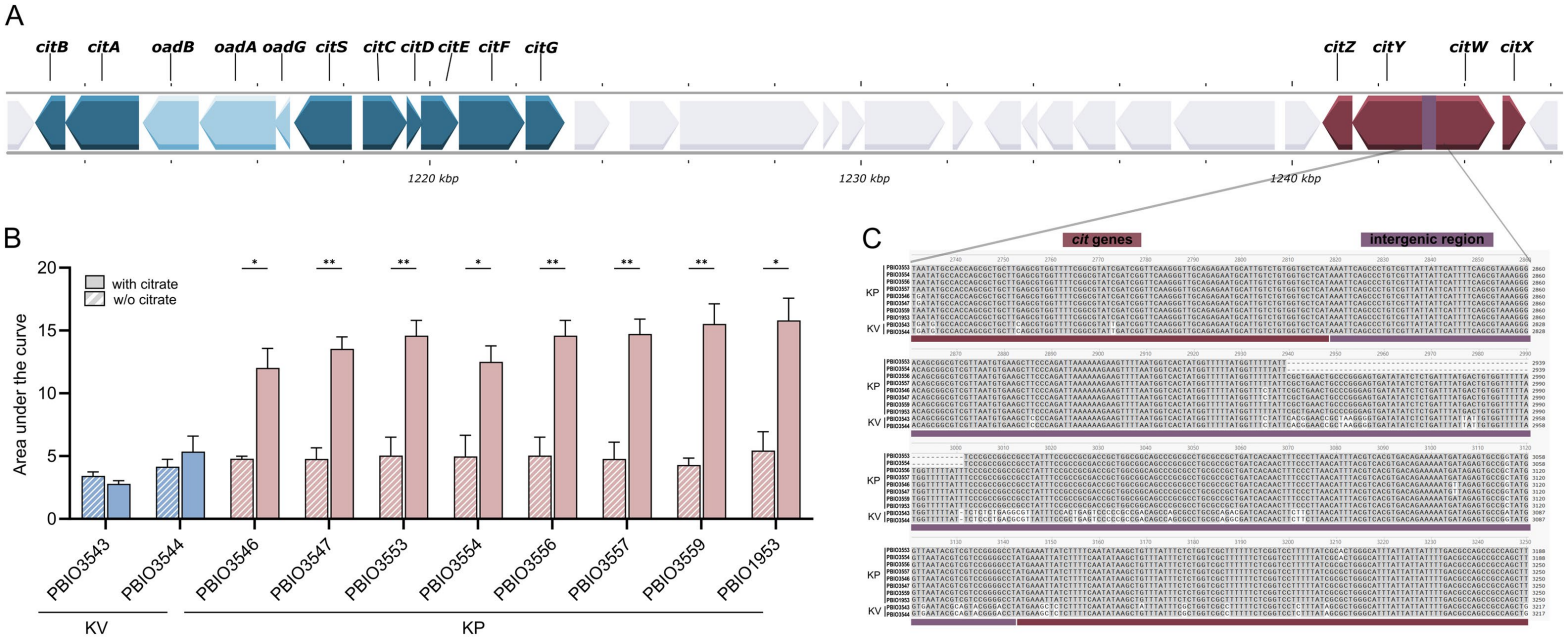
Genomic organization, growth impact, and sequence variation of citrate metabolism in *K. pneumoniae* (KP) and *K. variicola* (KV) strains. **(A)** Schematic representation of the two genomic regions encoding citrate metabolism genes in *K. pneumoniae*, visualized using Proksee. Genes involved in anaerobic citrate utilization are highlighted. **(B)** Strains were cultivated in synthetic human urine (SHU) with or without citrate supplementation in biological triplicates. The area under the curve (AUC) of growth kinetics for KP and KV strains cultivated in SHU with or without citrate supplementation was calculated from three independent cultures (*n* = 3). Significance of difference for each strain was assessed by unpaired parametric t-test using Bonferroni-Dunn correction for multiple comparisons (*p < 0.050; **p < 0.010). **(C)** Multiple sequence alignment of the *citWXYZ* genomic region, performed with Clustalw and visualized in SnapGene. The intergenic region (324 bp) of *citW* and *citY* is highlighted in boxes.

Citrate is not only a major component of the synthetic human urine but also abundant in urine, and strains encoding specific citrate uptake genes can utilize it as a sole carbon source under anaerobic conditions (Chen et al., 2009). To assess the impact of induced citrate metabolism, we modified SHU by depleting citrate and monitored the growth of both KP and KV strains. While this alteration had no noticeable effect on KV growth, it significantly reduced the growth of KP, bringing it down to KV levels (Figure 5B; Supplementary Figure 9). This finding underscores the functional importance of citrate utilization in KP and highlights its role as a specific fitness advantage under urine-like conditions. It has been shown that two genomic regions encoding genes for anaerobic citrate metabolism are encoded in *K. pneumoniae* genomes with remarkable genomic differences (Chen et al., 2009). Notably, we only detected KP-specific induction of one of the two regions (Figure 5A). Investigations of the present dataset revealed that the patterns of induced citrate metabolism and enhanced growth in presence of citrate did not correlate with either the presence of the one or two genomic regions, or with DGE of known regulators, e.g., CitB (response regulator), CitA (sensor kinase), or catabolite repressor protein (CRP). Expression of citrate genes is induced by CitB, which in turn stimulates CRP binding, consequently reduced CRP levels result in decreased gene expression (Meyer et al., 2001). Based on comparison with previously published CitB and CRP binding sites, we performed an multiple sequence alignment of KV and KP genomes (Meyer et al., 2001). We were able to detect pronounced sequence variation within potential CRP binding sites in the intergenic region of *citW* and *citY*, potentially explaining the different phenotypes (Figure 5C). Note that sequence variation within another part of the intergenic region was also detected for the KP ST147 isolates (PBIO3553 and PBIO3554). However, this did not affect the citrate-dependent phenotype.

The example of altered regulation of citrate genes demonstrates how a multi-omics approach is able to elucidate the impact of small genetic changes on transcriptomic and proteomic levels with significant relevance for the phenotypic appearance.

## 4 Discussion

Overall, multi-level omics studies have the potential to unveil adaptive and regulatory mechanisms of certain pathogens across various ecological niches, distinguishing them from more other, sometimes closely-related bacteria (O’Donnell et al., 2020; Mu et al., 2023). Despite the close relationship among members of the KpSC, KP stands out as the most prevalent and apparently successful member within the KpSC and the broader *Klebsiella* genus. It frequently serves as the causative agent of different infections, including UTIs (Wyres et al., 2020a; Rodríguez-Medina et al., 2024).

Here, we employed different levels of a multi-omics approach to understand the differences of *K. pneumoniae* when compared to *K. quasipneumoniae* and *K. variicola* (Wyres et al., 2020a; Lam et al., 2021; Gorrie et al., 2022; Murray et al., 2022). To the best of our knowledge, this study represents the first attempt to explore species-specific distinctions and pinpoint potentially novel markers specific to KP by leveraging genomic, transcriptomic, and proteomic data from a selection of clinical KpSC isolates, along with an extensive dataset comprising 6,740 publicly available whole genomes. We made a deliberate choice not to compare to rarer KP STs, as these are frequently linked to localized outbreaks despite their lower prevalence (Wyres et al., 2020a). In contrast, KV and KQ are rarely linked to outbreaks but still possess the capability to cause infections. Moreover, they are closely related to KP. Additionally, there was a correspondingly substantial number of genomes available for these species (Wyres et al., 2020a; Lam et al., 2021).

Our hypothesis posited that KP harbors distinct markers that are either absent or not induced in KV and KQ. These KP-specific markers may arise due to adaptive evolutionary processes undergone by successful KP lineages, resulting in core genes or gene functions that differ from those found in other bacteria (Holt et al., 2015; Wyres et al., 2020a). For the multi-omics analysis, KP isolates representing four distinct STs, which are members of the four most frequently detected KP clonal groups, were utilized (Wyres et al., 2020a). Indeed, our findings, combining growth kinetics in urine-like conditions with omics investigations, suggest that KP likely efficiently exploits available nutrients to subsequently induce additional metabolic pathways, resulting in enhanced growth and overall adaptation to this specific environment compared to KV. This conclusion is supported by the unique presence of various gene clusters categorized as GMs (e.g., *phn, pgt,* and *cel*) that are often associated with diverse metabolic pathways but also by the induction of the GMs and additional genes in urine-like conditions in KP but not KV (e.g., *cit*, and *cyd*). It is widely recognized that an adequate response and adaptation to specialized host environments, such as the urinary tract, are prerequisites for establishing subsequent infection (Alteri and Mobley, 2012; Martin and Bachman, 2018). Subsequent proteome and transcriptome analyses corroborated our initial observations. Not only did we identify similar regulatory patterns across all KP compared to KV strains, but our results also reinforce the notion that induced metabolic features enhance the ability to respond to specialized environments, such as the SHU tested in this study. These differentially expressed metabolic pathways included citrate acquisition, cytochrome ubiquinol oxidase, phosphorus source utilization, cellobiose utilization, and fatty acid biosynthesis, which appear to be crucial for general survival and growth (Wu et al., 2012; Giuffrè et al., 2014; Blin et al., 2017; Vornhagen et al., 2019).

A broad metabolic flexibility, as observed in KP, is essential for its capability to cause infections at multiple sites within hosts (Martin and Bachman, 2018). Such metabolic adaptation has been documented in extraintestinal *E. coli*, where they alter their metabolism from a commensal lifestyle in the intestine to a pathogenic one in the urinary tract (Alteri and Mobley, 2012). In addition to host colonization and adaptation, bacteria engage in competition with other microorganisms for nutrients, leading to varying preferences in the utilization of alternative nutrients (Alteri et al., 2015). Intriguingly, previous research has demonstrated that metabolic adaptation, facilitated by mechanisms like two-component systems, can trigger the expression of virulence-associated genes (Alteri and Mobley, 2012). For instance, we identified genes encoding for the cellobiose PTS across all omics levels, including the homologous clustering approach. This included the induction of the cellobiose PTS, including *celB*, which is known to play a role in bacterial biofilm formation and *in vivo* virulence in a mouse model of intragastric infection (Wu et al., 2012). The association between PTS and virulence has been previously demonstrated, such as the connection between the fructose PTS and fimbriae expression in *E. coli* (Rouquet et al., 2009). Also, the induction of the bacterial l-fucose metabolism seemingly promotes gastrointestinal colonization by KP, which increases the risk for a subsequent infection (Cano et al., 2022; Hudson et al., 2022).

We also identified markers that were present in KQ and KP genomes but absent in KV including genes for phosphorus metabolism (*phn and pgt*) that enable utilization of alternative phosphor sources, e.g., phosphonate (Kong et al., 2017; Poehlein et al., 2017). The broad occurrence of *phn* genes in clinical KP isolates, important for 2-aminoethylphosphonate transport and degradation, has already been reported (Ivan et al., 2014; Poehlein et al., 2017). Another example of the association between metabolism and virulence in KP under urine-like conditions is the *pgt-*operon, which is not only responsible for phosphoglycerate uptake via a two-component system but encoded on a pathogenicity island in uropathogenic *E. coli* together with the virulence-associated capsule (*kps*) locus (K15) (Podschun and Ullmann, 1998; Schneider et al., 2004). Further experiments are planned to investigate *phn* and *pgt* expression, among others, in KQ vs. KP in SHU.

In addition, we observed a KP-specific induction of the *cydAB* genes in SHU, despite their presence in all ten phenotypically characterized strains and in over 99.8% of the 6,740 KpSC. genomes. Apart from their role in generating proton motive forces, these genes have been associated with various other functions. This includes involvement in stress response, virulence, and evasion of the host immune system, as well as resistance to several classes of antimicrobials (Giuffrè et al., 2014).

Most interestingly, a gene cluster contributing to citrate utilization was found to be equally distributed among KV, KQ, and KP genomes, but only induced in KP in SHU. Note that citrate is abundant in urine, making this inducible response particularly relevant in UTIs (Sarigul et al., 2019). We provided phenotypic evidence supporting the physiological relevance of citrate utilization in KP. When citrate was depleted from the SHU, the growth advantage previously observed for KP over KV was markedly reduced, indicating that the induction of citrate-associated pathways contributes to the enhanced fitness of KP under these conditions. These examples illustrate that KpSC core genes undergo differential regulation when exposed to the same conditions. This regulation may be influenced by differences in regulatory transcripts and proteins or by genomic alterations in regulatory regions. We found evidence suggesting that sequence variation in KV compared to KP likely reduces the binding of regulatory proteins such as CRP. This reduction in binding appears to affect the activity of regulators, such as CitB, which in turn influences the expression of citrate-related genes (Meyer et al., 2001).

Moreover, prior studies have demonstrated that genes essential for citrate uptake not only confer the ability for citrate utilization but also serve in environmental sensing. The tight regulation of this process is facilitated by the two-component system CitAB (Reinelt et al., 2003). Bacterial cells adjust gene expression in response to environmental changes, especially concerning nutrient depletion and availability (Kinsella et al., 2011). Therefore, under *in vitro* conditions using a nutrient-defined synthetic urine medium, we expect bacteria to regulate genes involved in specialized metabolic pathways to optimize the use of available nutrients. However, it has also been commonly observed in *in vivo* settings that metabolic pathways significantly contribute to KP virulence and infection (Bachman et al., 2015; Ramos et al., 2018). For example, by using a transposon library, Bachman and colleagues elucidated key processes crucial for KP fitness in a lung infection model, pinpointing genes linked to bacterial metabolism, such as branched-chain amino acid metabolism (Bachman et al., 2015). The importance of metabolic flexibility, defined by the ability to acquire and utilize different metabolites, has also been demonstrated for mucosal surface colonization even prior to the establishment and manifestation of infection (Kinsella et al., 2011; Alteri and Mobley, 2012; Vornhagen et al., 2019). Vornhagen et al. showed that the citrate synthase GltA is essential for bacterial replication in the lung and intestine and mutation results in reduced metabolic flexibility including severe limitation of glycolic substrate utilization (Vornhagen et al., 2019). Our results suggest increased metabolic adaptation of KP vs. KV in urine-like conditions is not only limited to induction of citrate metabolism but a variety of metabolic pathways, which possibly contribute to the success of KP as an opportunistic pathogen. The flexible utilization of available nutrients can bolster the establishment of robust colonization, along with fimbrial attachment and capsule expression (Joseph et al., 2021). This, in turn, enhances the likelihood of subsequent infection (Gorrie et al., 2022).

It is evident that MDR and/or hypervirulent KP strains pose a significant threat to public health, necessitating urgent exploration of alternative treatment strategies (Geneva: World Health Organization, 2024). Our results offer valuable insights that could be integrated into further research in this area including differentiation of KP from KV and KQ in diagnostics approaches. Additionally, we have identified genes that may contribute to KP’s virulence potential in global dissemination. Alternative interventions, such as anti-virulence strategies targeting dysregulated metabolic pathways, hold promise (Murima et al., 2014). These approaches offer several advantages over conventional antibiotic therapy, including the availability of numerous (new) targets, reduced evolutionary pressure, minimal impact on the host commensal flora, and protection of immunocompromised individuals (Clatworthy et al., 2007; Dickey et al., 2017; Lau et al., 2023). Successful examples of alternative treatment strategies already exist, such as the inhibition of bacterial adhesion and colonization with mannosides in UTIs (Jarvisa et al., 2016) or the neutralization of toxins produced by *Clostridioides difficile* with antibodies (Lowy et al., 2010). Another example is targeting metabolic pathways, as demonstrated by trimethoprim’s inhibition of folate metabolism, resulting in bacterial stress due to the lack of essential metabolites required for DNA, RNA, and protein synthesis (Kwon et al., 2010). In our study, we uncovered the unique induction of the two-component system CitYZ homologous to CitAB in KP compared to KV. Disrupting the tight regulation, which senses and regulates citrate utilization, could potentially decrease KP fitness and prevent infection manifestation (Reinelt et al., 2003; Brisse et al., 2014). Another promising target for anti-virulence approaches is FabG, considering the potential of other proteins in the same pathway (e.g., FabI, FabB, FabH) as drug targets against KP (Ramos et al., 2018). These findings underscore the potential of the genes identified in our study, which warrant further investigation in subsequent studies.

In conclusion, our study offers significant insights into the genomics and adaptive responses of KP under urine-like conditions compared to KV, a closely related species within the KpSC. By investigating the expression of key genes and pathways unique to KP compared to other members of the KpSC, we contribute to the understanding of the complexity behind KP’s global prevalence and success. Our findings indicate that KP, in contrast to other KpSC species, possesses distinct genetic characteristics, and its regulation at the transcriptomic and proteomic levels is primarily focused on metabolic pathways. Ongoing prospective studies aim to explore whether the markers identified in this study can be utilized as novel druggable targets.

### 4.1 Limitations

There are some limitations to note. First, although our selection of KpSC species isolates provides a robust dataset, encompassing international high-risk clonal lineages of KP along with less prevalent KV and KQ, this selection was influenced by the availability of public genomic data. Transcriptomic and proteomic analyses in this study were limited to KP and KV, excluding KQ. To mitigate this limitation, homologous clustering including KQ was incorporated into the analysis to provide broader comparative context and minimize potential bias in the identification of KP-specific markers. Second, while KP is a major contributor to UTIs, its role extends to various other infectious contexts, and pathogenic strains may not necessarily enter through the urinary tract and the urinary tract may not always be the primary infection site. Despite this, the use of synthetic human urine remains a relevant model for simulating conditions found in critical KpSC infection sites. Third, functional validation of markers, such as through knock-out experiments, remains challenging, particularly with MDR isolates. Additionally, the sample size for transcriptomic and proteomic analyses is limited. Finally, while both KV and KQ show differences in the *citW–citY* intergenic region compared to KP, phenotypic validation and an increased sample size are required to make definitive conclusions about how these variations influence citrate utilization and contribute to KP’s apparent superiority.

## Data Availability

The original contributions presented in the study are publicly available. This data can be found here: https://www.ebi.ac.uk/ena, PRJEB71341; http://www.proteomexchange.org/, PXD047744.

## Footnotes

^1^ https://github.com/FelixKrueger/TrimGalore

^2^ https://github.com/FelixKrueger/TrimGalore

## Glossary

ACN: Acetonitrile
AMR: Antimicrobial resistance
BR: Biological replicates
cKp: Classical *K. pneumoniae*
COG: Cluster of orthologous groups
log_10_: Decimal logarithm
ESBL: Extended-spectrum *β*-lactamases
GM: Genomic markers from pangenome
GM-L: Genomic markers from homologous clustering
hvKp: Hypervirulent *K. pneumoniae*
iBAQ: Intensity-based absolute quantification
K: *Klebsiella*
KP: *Klebsiella pneumoniae*
KpSC: *Klebsiella pneumoniae* species complex
KQ: *Klebsiella quasipneumoniae*
KV: *Klebsiella variicola*
KEGG: Kyoto Encyclopedia of Genes and Genomes
L2FC: log2 fold change
MDR: Multidrug-resistant
MLST: Multi-locus sequence typing
NRC: Normalized read counts
OD_600_: Optical density of 0.05 at *λ* = 600 nm
PM: Proteomic markers
PTS: Phosphotransferase system
SDS: Sodium dodecyl sulfate
ST: Sequence type
SP3: Single pot solid-phase enhanced sample preparation
SHU: Synthetic human urine
TM: Transcriptomic markers
UTI: Urinary tract infection
