# Supplementary material for: Multi-omics investigation reveals unique markers in Klebsiella pneumoniae compared to closely related species
Source: Front Microbiol. 2025 Aug 20;16:1657680. doi: 10.3389/fmicb.2025.1657680 (PMC12405260; doi:10.3389/fmicb.2025.1657680)
Supplement: Supplementary file 1 [file Supplementary_file_1.pdf]

Supplement

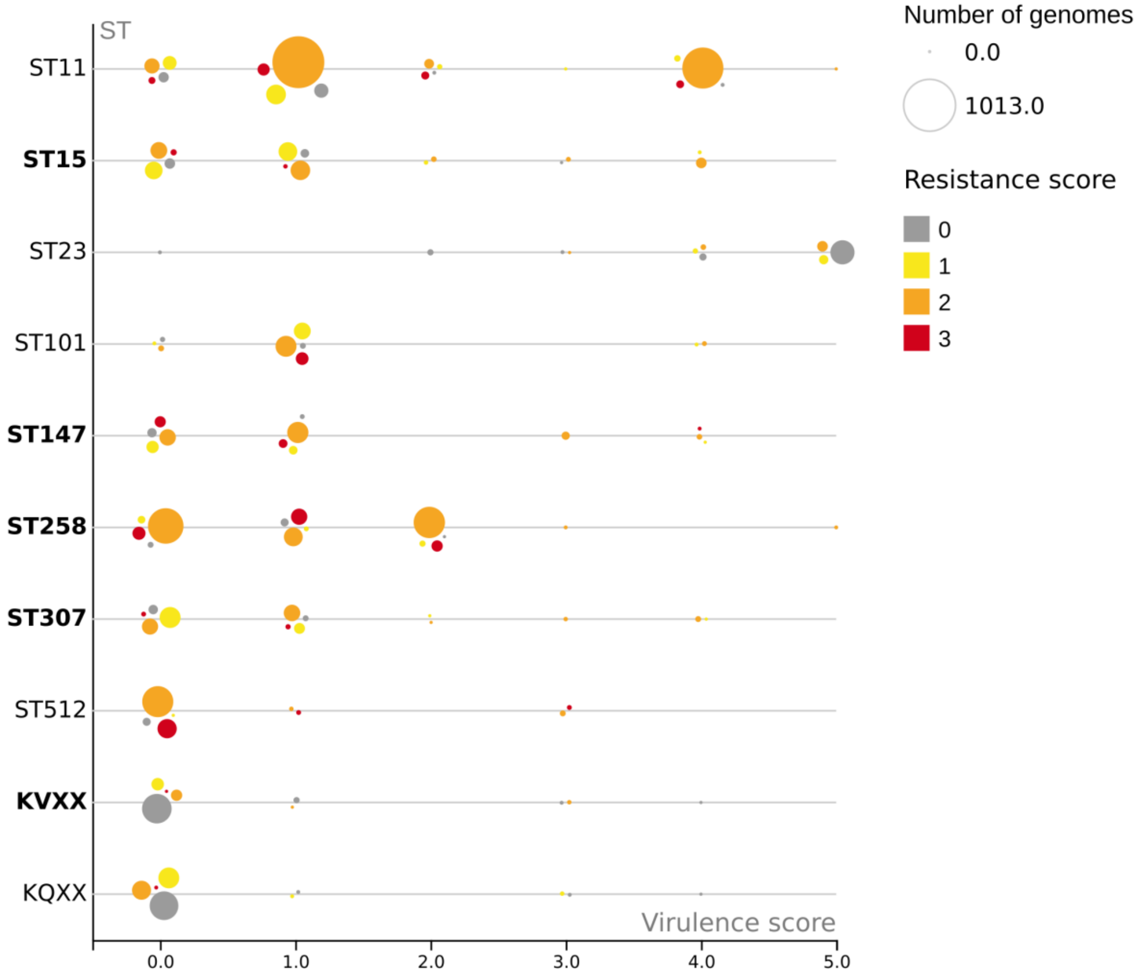

Supplementary figure 1: **Beeswarm plot of the virulence and resistance scores of *K. pneumoniae* (KP), *K. variicola* (KV) *and K. quasipneumoniae* (KQ).** The resistance score (0: no ESBL, no carbapenemases (CARB) (regardless of colistin resistance), 1: ESBL, no CARB (regardless of colistin resistance), 2: CARB without colistin resistance (regardless of ESBL genes or OmpK mutations), 3: CARB with colistin resistance (regardless of ESBL genes or OmpK mutations)) and the virulence score (0=negative for all of yersiniabactin (*ybt*), colibactin (*clb*), aerobactin (*iuc*), 1= *ybt* only, 2= *ybt* and *clb* (or *clb* only), 3=aerobactin (without *ybt* or *clb*), 4=aerobactin with *ybt* (without *clb*), 5= *ybt*, *clb* and *iuc*) were determined for each of the included genomes (n=6,740) using Kleborate v.2.2.3. Raw Kleborate output can be found in Supplementary table 5. The different KP sequence types (STs), as well as the pool of KV and KQ STs, indicated by KVXX and KQXX, are plotted on the x-axis. The size of the bubbles indicates the number of genomes with the respective Kleborate resistance score that is represented by the color of the bubbles. The virulence score is plotted on the y-axis. KP STs as well as KV included in the pangenome approach are highlighted in bold.

Supplementary table 1: Instrumental setting for Reversed phase liquid chromatography (RPLC) and mass spectrometry.

	Reversed phase liquid chromatography (RPLC)
Instrument	Ultimate 3000 RSLC (Thermo Scientific)
Trap column	75 µm inner diameter, packed with 3 µm C18 particles (Acclaim PepMap100, Thermo Scientific)
Analytical column	Accucore 150-C18, (Thermo Fisher Scientific)<br>25 cm x 75 µm, 2.6 µm C18 particles, 150 Å pore size
Buffer system	binary buffer system consisting of 0.1% acetic acid in HPLC-grade water (buffer A) and 100% ACN in 0.1% acetic acid (buffer B)
Flow rate	300 nl/min
Gradient	0 min 2% B →<br>2 min 5% B →<br>10 min 5% B →<br>130 min 25% B →<br>135 min 40% B →<br>137 min 90% B →<br>142 min 90% B →<br>145 min 2% B →<br>150 min 2% B
Gradient duration	120 min
Column oven temperature	40°C
	Mass spectrometry
Instrument	Q Exactive HF mass spectrometer
Electrospray	Nanospray Flex Ion Source
Operation mode	data-independent
Full MS	
MS scan resolution	60000
Norm. AGC target	5e6
maximum ion injection time for the MS scan	200 ms
Scan range	333 to 1650 m/z
RF Lens	50%
Spectra data type	profile
dd-MS2	
Precursor mass range	333 to 1650 m/z
Resolution	30,000
Norm. MS/MS AGC target	3e6
Maximum ion injection time mode	auto
Spectra data type	profile
Microscans	1
Isolation window	56 windows, 13 m/z, 2 m/z overlap
Define first mass	200
Dissociation mode	higher energy collisional dissociation (HCD)
Normalized collision energy	27.5 %

Supplementary table 2: **Spectronaut settings for mass spectrometry**.

ANALYSIS DATA
Spectronaut 18.1.230626.50606
Analysis Mode: UI
Analysis Type: directDIA
Analysis Date: 27-July-2023 10:50:27 UTC+0
BEGIN-SETTINGS
Settings Used: C_FunGene_directDIA_sparse_no_imputing
├── DIA Analysis\Calibration
│ ├── MZ Extraction Strategy: Maximum Intensity
│ ├── Allow source specific iRT Calibration: True
│ ├── Precision iRT: True
│ │ ├── Exclude De-amidated Peptides: True
│ │ └── iRT <-> RT Regression Type: Local (Non-Linear) Regression
│ ├── MS1 Mass Tolerance Strategy: System Default
│ └── MS2 Mass Tolerance Strategy: System Default
├── DIA Analysis\Identification
│ ├── Precursor Qvalue Cutoff: 0.001
│ ├── Precursor PEP Cutoff: 0.2
│ ├── Protein Qvalue Cutoff (Experiment): 0.01
│ ├── Protein Qvalue Cutoff (Run): 0.05
│ ├── Protein PEP Cutoff: 0.75
│ ├── Single Hit Definition: By Stripped Sequence
│ ├── Exclude Single Hit Proteins: False
│ ├── Exclude Duplicate Assays: True
│ ├── Exclude Predicted Fragment Scores: False
│ ├── Generate Decoys: True
│ │ ├── Decoy Generation Method: Mutated
│ │ │ └── Preferred Fragment Source: NN Predicted Fragments
│ │ └── Decoy Limit Strategy: Dynamic
│ │ └── Library Size Fraction: 0.1
│ └── Pvalue Estimator: Kernel Density Estimator
├── DIA Analysis\Pipeline Mode
│ ├── Generate SNE File: True
│ │ └── Store Ion traces in SNE: False
│ ├── Post Analysis Reports:
│ │ ├── CV Density Line Chart: True
│ │ ├── CVs Below X Bar Chart: True
│ │ ├── Data Completeness Bar Chart: True
│ │ ├── Run Identifications Bar Chart: True
│ │ └── Scoring Histograms: True
│ ├── Report Schema: C_FunGene_complex (Normal), C_FunGene_complex (Normal)
│ └── Reporting Unit: Across Experiment
├── DIA Analysis\Post Analysis
│ ├── Differential Abundance Testing: Paired t-test
│ │ ├── Group-Wise Testing Correction: False
│ │ ├── Log2 Ratio Candidate Filter: 0.58
│ │ └── Confidence Candidate Filter: Qvalue
│ │ └── Confidence: 0.05
│ ├── Differential Abundance Grouping: Major Group (Quantification Settings)
│ │ └── Smallest Quantitative Unit: Precursor Ion (Quantification Settings)
│ │ └── Use All MS-Level Quantities: False
│ ├── Calculate Explained TIC: None
│ ├── Calculate Sample Correlation Matrix: True
│ └── Hierarchical Clustering: True
│ ├── Distance Metric: Manhattan Distance
│ ├── Linkage Strategy: Ward's Method
│ ├── Order Runs by Clustering: True
│ └── Z-score Transformation: False
├── DIA Analysis\Protein Inference
│ └── Protein Inference Workflow: Automatic
│ └── Inference Algorithm: IDPicker
├── DIA Analysis\PTM Workflow
│ └── PTM Localization: False
├── DIA Analysis\Quantification
│ ├── Precursor Filtering: Identified (Qvalue)
│ │ └── Imputation Strategy: Use Background Signal

```
│  ├─ Proteotypicity Filter:    None
│  ├─ Protein LFQ Method:    MaxLFQ
│  ├─ Quantity MS Level:    MS2
│  ├─ Quantity Type:    Area
│  ├─ Cross-Run Normalization:    True
│  │  ├─ Normalization Filter Type:    None
│  │  ├─ Normalization Strategy:    Local Normalization
│  │  └─ Row Selection:    Identified in at least 1 Run (Sparse)
│  ├─ Quantification window: Not Synchronized (SN 17)
│  ├─ Interference Correction:True
│  │  ├─ Only Identified Peptides:    True
│  │  ├─ Exclude All Multi-Channel Interferences:    True
│  │  ├─ MS1 Min:    2
│  │  └─ MS2 Min:    3
│  ├─ Major (Protein) Grouping:    by Protein Group Id
│  ├─ Minor (Peptide) Grouping:    by Stripped Sequence
│  ├─ Major Group Quantity: Mean peptide quantity
│  ├─ Major Group Top N:    True
│  │  ├─ Max:    3
│  │  └─ Min:    2
│  ├─ Minor Group Quantity: Sum precursor quantity
│  └─ Minor Group Top N:    False
├─ DIA Analysis\Workflow
│  ├─ Method Evaluation:    False
│  ├─ MS2 DeMultiplexing:    Automatic
│  ├─ Profiling Strategy:    iRT Profiling
│  │  ├─ Carry-over exact Peak Boundaries:    False
│  │  ├─ Profiling Row Selection:    Minimum Qvalue Row Selection
│  │  │  └─ Qvalue Threshold:    0.001
│  │  └─ Profiling Target Selection:    Profile only non-identified Precursors
│  │     ├─ Identification Criterion:    Qvalue
│  │     └─ Threshold:    0.001
│  ├─ Run Limit for directDIA Library:    -1
│  └─ Unify Peptide Peaks Strategy:    Select corresponding Peak
├─ DIA Analysis\XIC Extraction
│  ├─ XIC IM Extraction Window:    Dynamic
│  │  └─ Correction Factor:    1
│  ├─ XIC RT Extraction Window:    Dynamic
│  │  └─ Correction Factor:    1
│  ├─ MS1 Mass Tolerance Strategy:    Dynamic
│  │  └─ Correction Factor:    1
│  └─ MS2 Mass Tolerance Strategy:    Dynamic
│     └─ Correction Factor:    1
├─ Pulsar Search\Identification
│  ├─ PSM FDR:    0.01
│  ├─ Peptide FDR:    0.01
│  ├─ Protein Group FDR:    0.01
│  ├─ directDIA Workflow:    directDIA+ (Deep)
│  └─ PTM Localization Filter:    False
├─ Pulsar Search\Labeling
│  └─ Channels:
│     ├─ Channel 1:False
│     ├─ Channel 2:False
│     └─ Channel 3:False
├─ Pulsar Search\Modifications
│  ├─ Max Variable Modifications:    5
│  └─ Select Modifications:
│     ├─ Fixed Modifications::
│     └─ Variable Modifications: :    Oxidation (M)
├─ Pulsar Search\Peptides
│  ├─ Enzymes / Cleavage Rules:    Trypsin/P
│  ├─ Digest Type:Specific
│  ├─ Max Peptide Length:    52
│  ├─ Min Peptide Length:    7
│  ├─ Missed Cleavages:    0
│  └─ Toggle N-terminal M:    True
├─ Pulsar Search\Result Filters
```

```
│  ├─ Fragment Ions:
│  │  ├─ Ion AA Length:       True
│  │  │  └─ N:      3
│  │  ├─ Ion Charge:          False
│  │  ├─ Ion Loss Type:       False
│  │  ├─ Ion Type: False
│  │  ├─ m/z :      True
│  │  │  ├─ Max:   1800
│  │  │  └─ Min:   300
│  │  └─ Relative Intensity:  True
│  │    └─ Min:    5
│  └─ Precursors:
│    ├─ Amino Acids:          False
│    ├─ Best N Fragments per Peptide:    True
│    │  ├─ Max:   10
│    │  └─ Min:   6
│    ├─ Best N Peptides per Protein Group:         False
│    ├─ Channel Count:        False
│    ├─ FASTA Matched:        False
│    ├─ Missed Cleavage:      False
│    ├─ Modifications:        None
│    ├─ Peptide Charge:       False
│    └─ Proteotypicity:       False
├─ Pulsar Search\Speed-Up
│  ├─ IM DFD Processing:
│  │  └─ Use Dynamic IM Peak Filter:   True
│  │    └─ Target TIC Fraction:        0.9
│  └─ MS2 Index:  Automatic
├─ Pulsar Search\Tolerances
│  └─ Tolerance Parameters:
│    ├─ Thermo IonTrap:
│    │  ├─ Calibration Search:         Dynamic
│    │  │  ├─ MS1 Correction Factor:   1
│    │  │  └─ MS2 Correction Factor:   1
│    │  └─ Main Search:       Dynamic
│    │    ├─ MS1 Correction Factor:    1
│    │    └─ MS2 Correction Factor:    1
│    ├─ Thermo Orbitrap:
│    │  ├─ Calibration Search:         Dynamic
│    │  │  ├─ MS1 Correction Factor:   1
│    │  │  └─ MS2 Correction Factor:   1
│    │  └─ Main Search:       Dynamic
│    │    ├─ MS1 Correction Factor:    1
│    │    └─ MS2 Correction Factor:    1
│    └─ TOF:
│      ├─ Calibration Search:  Dynamic
│      │  ├─ MS1 Correction Factor:    1
│      │  └─ MS2 Correction Factor:    1
│      └─ Main Search:        Dynamic
│        ├─ MS1 Correction Factor:     1
│        └─ MS2 Correction Factor:     1
└─ Pulsar Search\Workflow
  ├─ Fragment Ion Selection Strategy:   Intensity Based
  ├─ In-Silico Generate Missing Channels:        False
  └─ Use DNN Predicted Ion Mobility:   Auto
```

END-SETTINGS

Supplementary table 3 provided as separate data file (Supplementary file 2.xlsx):

Supplementary table 3: **Summary of clusters generated for the pangenome analysis of *K. pneumoniae* (KP) and *K. variicola* (KV).** Each cluster is listed with its respective cluster ID. Further, the table contains information on the annotation of each cluster together with the number of species it was detected in. In addition, a presence and absence matrix is shown for the individual strains, whereas presence of the cluster within the strain's genome is indicated with "yes" and absence with "no" Finally, the category the cluster belongs to is shown as "core" = clusters shared by all strains, "shell" = clusters present in a minimum of two and a maximum of nine strains, "cloud" = clusters unique to a single genome within the dataset, or "KP-marker" = clusters shared by all KP strains but absent in KV strains.

Supplementary table 4 provided as separate data file (Supplementary file 3.xlsx):

Supplementary table 4: **Summary of transcriptome and proteome.** Each cluster within the transcriptome and proteome is identified by a unique cluster ID (cluster ID pangenome). Annotation and Prokka ID are shown for the respective representative cluster ID for the pangenome. The cluster IDs from the homologous clustering approach are assigned to the cluster ID of the pangenome for each strain and the representative cluster ID is shown (association of clusters from pangenome and homologous clustering approach). The relative occurrence of the cluster within the homologous clustering approach is shown for each sequence type (ST) of *K. pneumoniae* (KP), *K. variicola* (KV) and *K quasipneumoniae* (KQ) (genome). The L2FC data obtained from the normalized read counts (NRC) are shown for all comparisons (transcriptome). In addition, the log2 fold change (L2FC) calculated from the tensity-based absolute quantification (iBAQ) (proteome) are shown for all clusters.

Supplementary table 5 provided as separate data file (Supplementary file 3.xlsx):

Supplementary table 5: **Accession numbers and Kleborate results for the 6,740 genomes.** The table includes accession numbers and the Kleborate output for all the genomes included in the homologous clustering and in the pangenome. The column "Pangenome/Transcriptome/Proteome" indicates whether a strain/genome was included in the pangenome, transcriptome and proteome approach ("yes") or in the homologous clustering only ("no"). If the genomes have been included in the pangenome, strain name and the accession number from Nanopore sequencing is additionally shown. The raw output of the Kleborate analysis for obtaining resistance and virulence score is shown for each genome (Kleborate v.2.2.3).

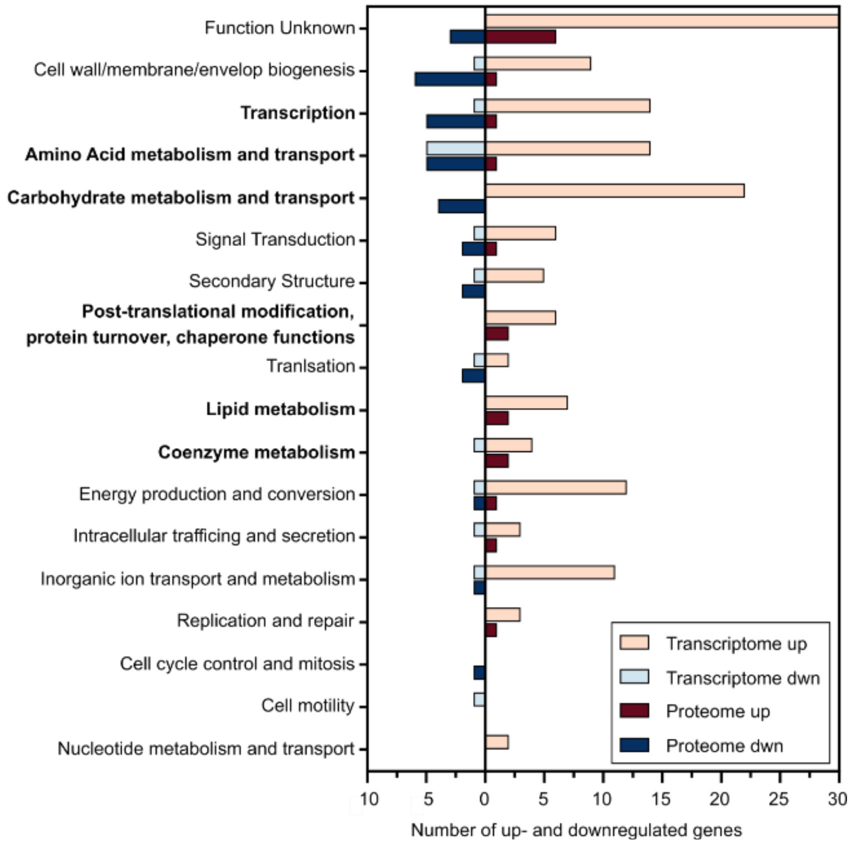

Supplementary figure 2: **Number of up- and downregulations on transcriptomic and proteomic levels assigned to the different cluster of orthologous groups (COG) categories.** Whenever available, information about the COG for all regulated genes on transcriptomic and proteomic levels were annotated based on the Bakta and eggNOG database and plotted separately on transcriptomic and proteomic levels and up- or down-regulated genes as indicated.

Supplementary table 6: **Functional categories designed for in-depth characterization of regulated genes with their respective numbers**. Four different main groups (Porins/transporter, metabolism, regulation, and others) were assigned. In a second step, more detailed categories were defined. Number of regulations were differentiated between proteomic and transcriptomic levels and whether they are up- (up) or down (dwn) regulated

Category number	Category	Proteome dwn	Proteome up	Transcriptome dwn	Transcriptome up
**Porins/transporter**					
**1**	Porin	0	1	0	3
**2**	ABC transporter	5	0	1	6
**3**	PTS	0	0	0	0
**4**	Expoter	1	0	0	0
**5**	MFS transporter	0	0	2	5
**6**	Permease	0	0	0	2
**7**	Two component system	0	0	0	4
**8**	other transporter	0	0	0	4
**Metabolism**					
**9**	Sulfur	0	1	0	8
**10**	Nitrogen/ammonia	0	1	0	2
**11**	Citrate	0	3	0	2
**12**	Posphate	0	0	0	7
**13**	Biotin/fatty acid	0	2	1	5
**14**	Carbohydrate	3	2	2	17
**15**	Amino acid	3	1	0	18
**16**	O-antigen/LPS/cell well	4	0	0	6
**17**	Ribosomes/tRNA	2	1	0	3
**18**	Peptide	0	2	0	3
**19**	Nucleotide	0	0	0	1
**Regulation/stress response**					
**20**	regulators / transcription factors	5	1	1	12
**21**	stress response	0	1	0	4
**others**					
**22**	chaperones	0	0	0	2
**23**	toxin-antitoxin	1	0	0	1
**24**	DNA	0	1	0	3
**25**	unkown	0	2	2	9
**26**	resistance	2	0	1	0
**27**	others	3	1	2	17
**total number**		**29**	**20**	**12**	**144**

Supplementary table 7: **Assignment of clusters to the developed functional category.** Each cluster was assigned to a category number (Supplementary table 5). Further it is differentiated whether up- or down-regulation in *K. pneumoniae* compared to *K. variicola* was detected on transcriptomic (RNA) or proteomic (protein) level.

Cluster ID	Category number	Regulation	Omics level
cl00019	10	up	protein
cl00122	1	up	RNA
cl00156	9	up	RNA
cl00275	27	down	protein
cl00362	21	up	RNA
cl00441	2	up	RNA
cl00452	10	up	RNA
cl00491	27	up	RNA
cl00571	13	up	protein
cl00690	15	up	RNA
cl00734	5	down	RNA
cl00851	14	up	RNA
cl01016	14	down	RNA
cl01020	2	up	RNA
cl01035	27	up	RNA
cl01080	15	down	protein
cl01435	5	down	RNA
cl01457	21	up	RNA
cl01457	21	up	protein
cl01478	14	up	RNA
cl01483	27	down	RNA
cl01520	11	up	RNA
cl01520	11	up	protein
cl01595	21	up	RNA
cl01810	9	up	RNA
cl01855	20	up	RNA
cl01884	11	up	RNA
cl01884	11	up	protein
cl01934	24	up	RNA
cl01978	9	up	RNA
cl02041	11	up	protein
cl02165	2	up	RNA
cl02168	17	up	protein
cl02199	27	up	RNA
cl02200	20	up	RNA
cl02207	27	down	RNA
cl02262	14	down	RNA
cl02455	22	up	RNA
cl02465	14	up	RNA
cl02470	20	up	RNA
cl02761	2	down	protein
cl02771	20	down	protein
cl02871	25	down	RNA
cl02893	14	up	RNA
cl02930	9	up	RNA
cl02931	9	up	RNA
cl02947	17	down	protein
cl02948	17	up	RNA
cl02956	1	up	RNA
cl02956	1	up	protein
cl02960	13	down	RNA
cl03037	20	down	RNA
cl03041	18	up	RNA
cl03093	2	up	RNA
cl03182	2	down	RNA
cl03246	25	down	RNA
cl03293	27	up	RNA
cl03297	9	up	protein
cl03396	9	up	RNA
cl03525	20	down	protein
cl03537	4	down	protein
cl03542	14	down	protein
cl03634	23	down	protein
cl03745	7	up	RNA
cl03746	7	up	RNA
cl03828	24	up	protein
cl03864	18	up	RNA
cl03864	18	up	protein
cl03866	18	up	protein
cl03944	18	up	RNA
cl03962	26	down	RNA
cl03973	17	down	protein
cl03976	20	up	protein
cl04012	15	up	RNA
cl04087	14	up	RNA
cl04088	2	down	protein
cl04110	27	up	protein
cl04243	8	up	RNA
cl04244	5	up	RNA
cl04278	12	up	RNA
cl04306	20	up	RNA
cl04307	15	up	RNA
cl04309	25	up	RNA
cl04310	20	up	RNA
cl04310	20	up	protein
cl04311	13	up	RNA
cl04312	13	up	RNA
cl04315	15	up	RNA
cl04316	20	up	RNA
cl04321	27	up	RNA
cl04322	14	up	RNA
cl04322	14	up	protein
cl04323	8	up	RNA
cl04324	25	up	RNA
cl04325	22	up	RNA
cl04326	10	up	RNA
cl04327	16	up	RNA
cl04328	25	up	RNA
cl04329	15	up	RNA
cl04332	27	up	RNA
cl04332	27	up	protein
cl04333	5	up	RNA
cl04334	13	up	RNA
cl04334	13	up	protein
cl04335	25	up	RNA
cl04336	5	up	RNA
cl04337	6	up	RNA
cl04337	6	up	RNA
cl04341	16	up	RNA
cl04342	23	up	RNA
cl04343	20	up	RNA

Cluster ID	Category number	Regulation	Omics level	Cluster ID	Category number	Regulation	Omics level
cl04344	20	up	RNA	cl04472	7	up	RNA
cl04347	27	up	RNA	cl04473	27	up	RNA
cl04348	20	up	RNA	cl04474	14	up	RNA
cl04349	19	up	RNA	cl04475	20	up	RNA
cl04351	20	up	RNA	cl04476	5	up	RNA
cl04352	21	up	RNA	cl04477	27	up	RNA
cl04353	15	up	RNA	cl04478	5	up	RNA
cl04356	27	up	RNA	cl04479	12	up	RNA
cl04361	24	up	RNA	cl04481	17	up	RNA
cl04362	15	up	RNA	cl04482	7	up	RNA
cl04364	24	up	RNA	cl04484	12	up	RNA
cl04365	16	up	RNA	cl04485	15	up	RNA
cl04366	25	up	RNA	cl04486	14	up	RNA
cl04368	15	up	RNA	cl04487	15	up	RNA
cl04370	20	up	RNA	cl04487	15	up	protein
cl04371	15	up	RNA	cl04490	17	up	RNA
cl04374	27	up	RNA	cl04491	27	up	RNA
cl04376	1	up	RNA	cl04500	15	up	RNA
cl04377	14	up	RNA	cl05041	26	down	protein
cl04377	14	up	protein	cl05158	27	down	protein
cl04379	12	up	RNA	cl06254	16	down	protein
cl04380	15	up	RNA	cl06255	16	down	protein
cl04381	27	up	RNA	cl06256	16	down	protein
cl04382	27	up	RNA	cl06257	16	down	protein
cl04383	14	up	RNA	cl06295	2	down	protein
cl04385	27	up	RNA	cl06305	14	down	protein
cl04386	16	up	RNA	cl06336	25	down	protein
cl04390	15	up	RNA	cl06349	14	down	protein
cl04391	20	up	RNA	cl06382	2	down	protein
cl04392	16	up	RNA	cl06385	2	down	protein
cl04394	8	up	RNA	cl06388	26	down	protein
cl04403	27	up	RNA	cl06393	15	down	protein
cl04407	15	up	RNA	cl06417	20	down	protein
cl04416	25	up	RNA	cl06439	20	down	protein
cl04418	16	up	RNA	cl06450	15	down	protein
cl04419	27	up	RNA	cl06452	20	down	protein
cl04422	14	up	RNA				
cl04423	2	up	RNA				
cl04428	25	up	RNA				
cl04433	12	up	RNA				
cl04435	25	up	RNA				
cl04439	12	up	RNA				
cl04442	14	up	RNA				
cl04444	14	up	RNA				
cl04445	15	up	RNA				
cl04447	15	up	RNA				
cl04448	13	up	RNA				
cl04451	9	up	RNA				
cl04452	2	up	RNA				
cl04453	9	up	RNA				
cl04456	12	up	RNA				
cl04459	13	up	RNA				
cl04460	14	up	RNA				
cl04463	14	up	RNA				
cl04466	8	up	RNA				
cl04469	14	up	RNA				
cl04470	15	up	RNA				
cl04471	14	up	RNA				

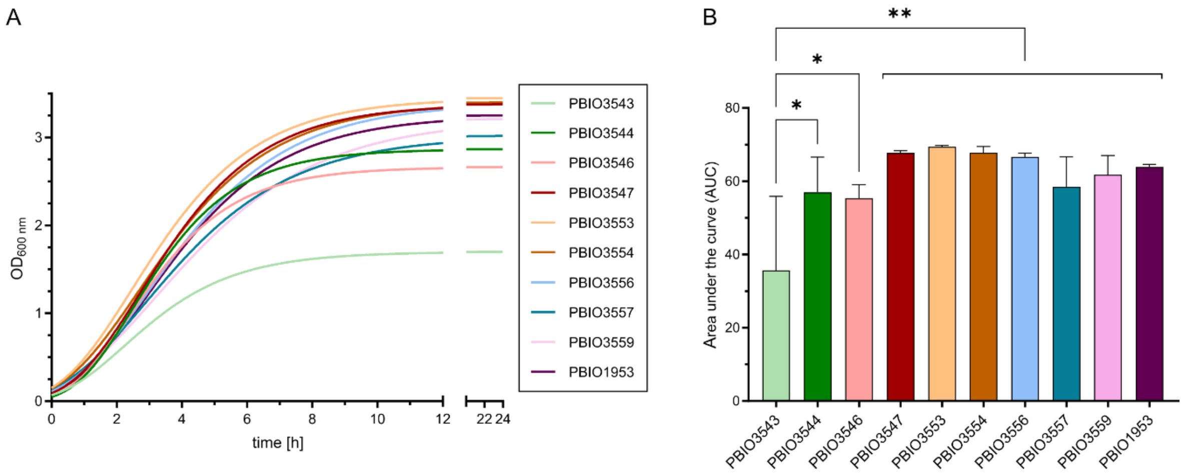

Supplementary figure 3: **Growth kinetics of *K. pneumoniae* (KP) and *K. variicola* (KV) strains in LB medium.** Strains were cultivated in LB medium in biological triplicates. (**A**) Growth kinetics are displayed using GraphPad Prism and Gompertz-Growth fitting. (**B**) The area under the curve was calculated from three individual cultivations. The significance of differences was calculated using one-way ANOVA (**p* <0.05; ***p* <0.01; ****p* <0.001).

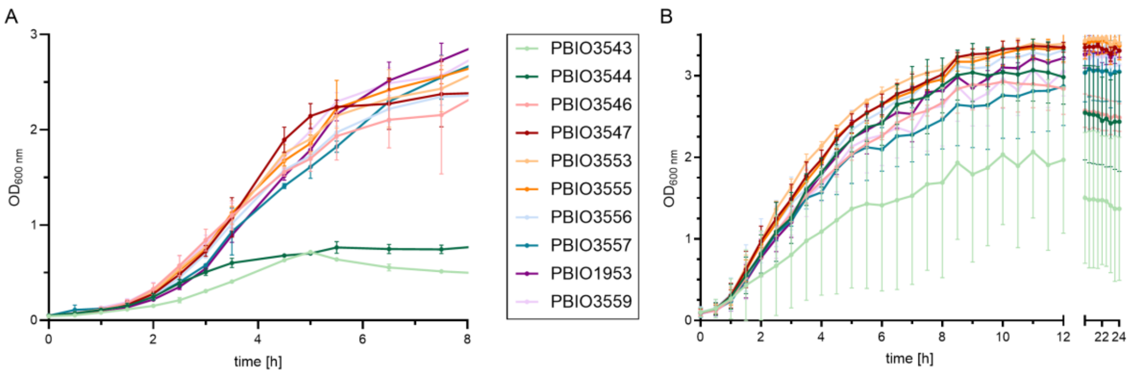

Supplementary figure 4: **Growth kinetics used for Gompertz-Growth fitting:** Data was plotted from indicated strains (n=3) grown in (**A** synthetic human urine and (**B**) LB medium. Mean values and standard deviations are shown.

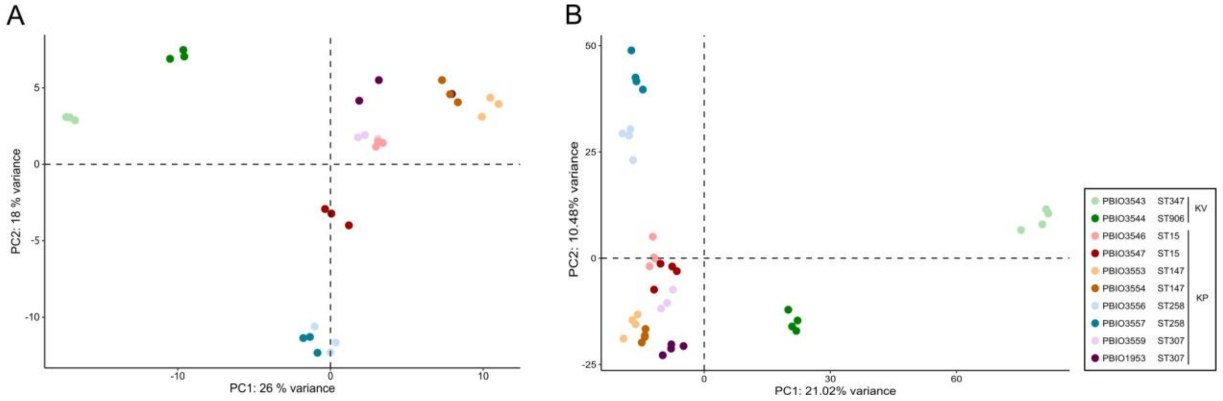

Supplementary figure 5: **Principle component (PC) analysis.** (**A**) Transcriptomic samples were separated by the PC1 (26% variance) and PC2 (18% variance). Data from three biological replicates are represented. (**B**) Proteomic samples were separated by the PC1 (21.02% variance) and PC2 (10.48% variance). Data from minimum three biological replicates are represented.

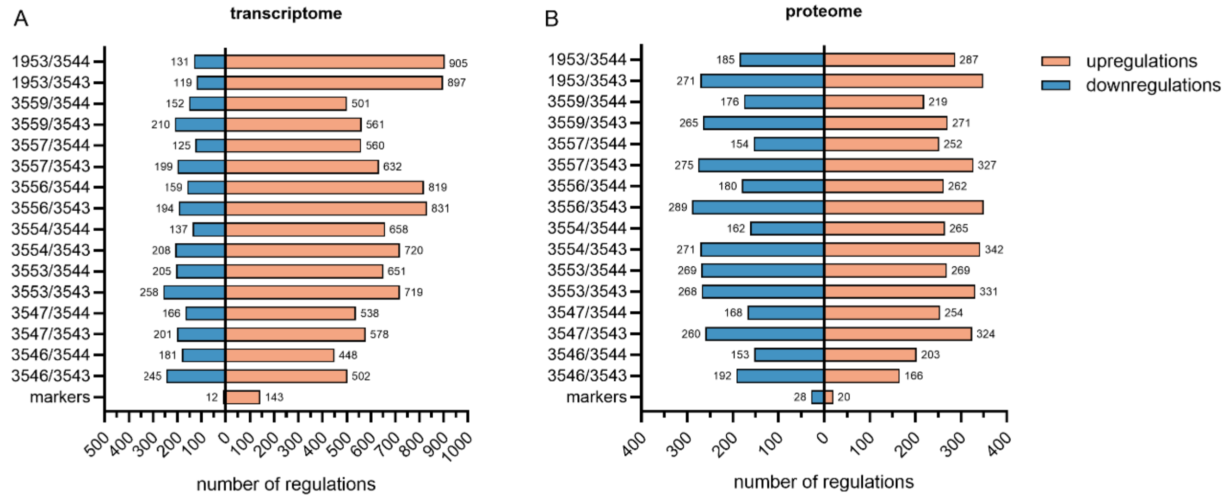

Supplementary figure 6: **Number of strain specific and shared regulations (markers) on transcriptomic and proteomic levels.** Differentially expressed genes and proteins abundancies were analyzed by comparing each *K. pneumoniae* (KP) strain with each *K. variicola* (KV) strain. Genes were assumed as differentially expressed or proteins as significantly different in their abundance when the comparison revealed a |L2FC|>1.5 and a significant *p*-adjusted value of <0.05. Depending on the L2FC, it differentiated between downregulations (L2FC<-1.5) or upregulations (L2FC>1.5). The x-axis shows the number of differentially expressed genes (**A**) and significant differences in protein abundance (**B**) for each comparison shown on the y-axis. Note that the comparisons are shown as the shortened strain IDs of strain 1 compared to strain 2 (strain 1/strain 2). Additionally, the number of markers is indicated, which comprises regulations of the genes or proteins shared among KP in contrast to KV.

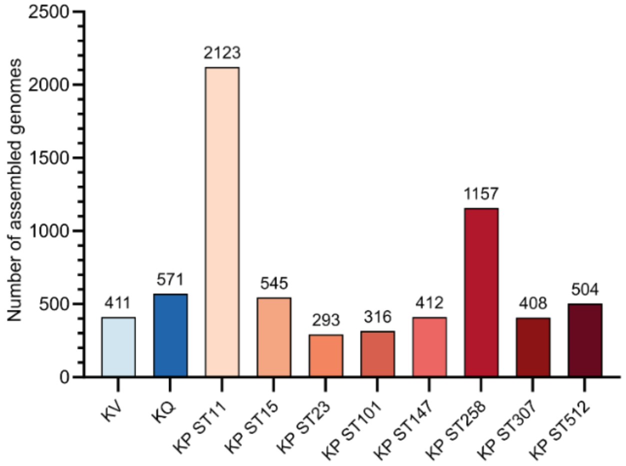

Supplementary figure 7: **Number of publicly available genomes used for the construction of a *K. pneumoniae* species complex homologous clustering approach.** The number of genomes downloaded from the NCBI database is shown for *K. variicola* (KV), *K. quasipneumoniae* (KQ) and each of the 8 successful international high-risk *K. pneumoniae* (KP) sequence type (ST).

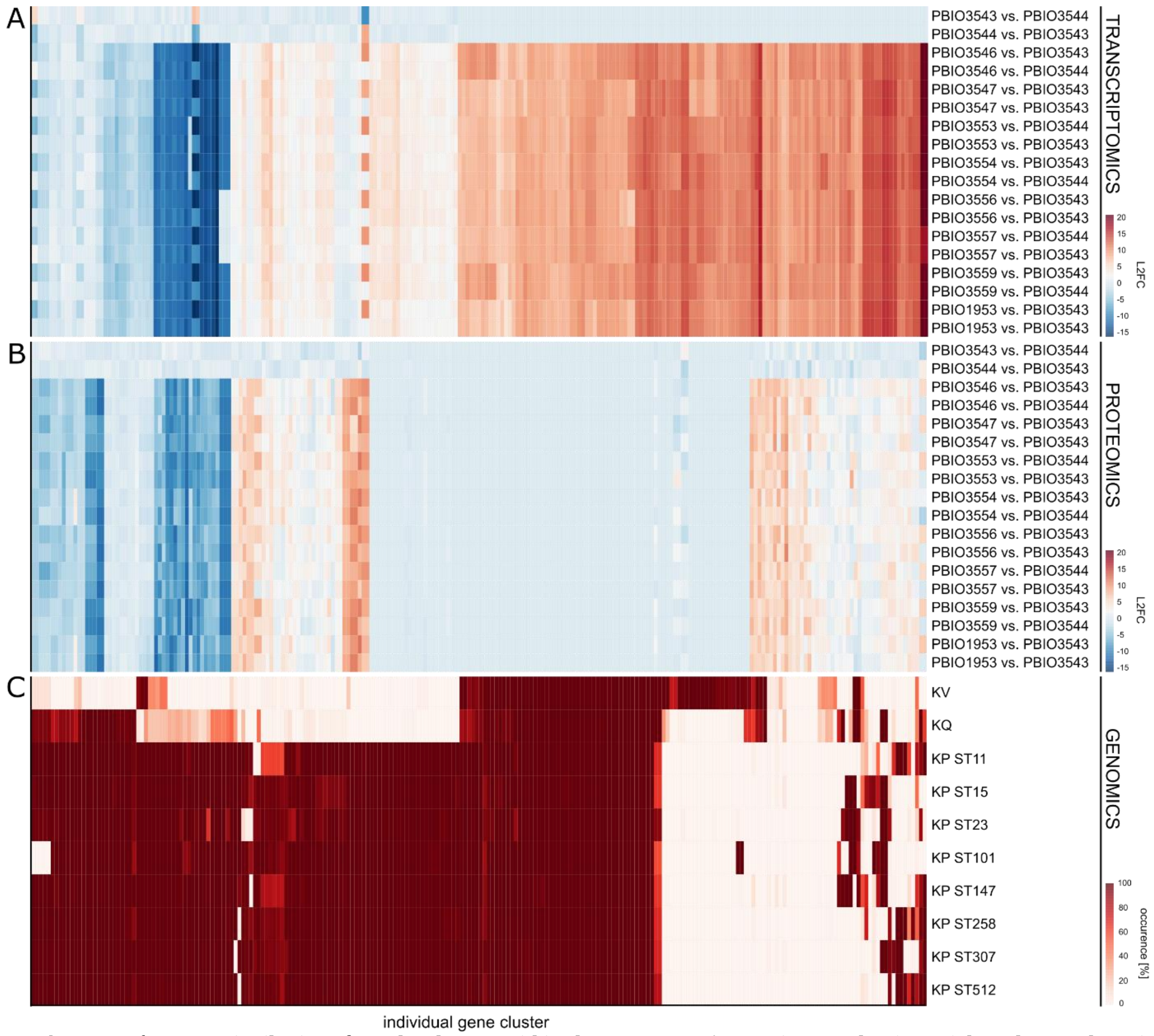

Supplementary figure 8: **Distribution of regulated genes within the *K. pneumoniae* species complex (KpSC) homologous clustering approach**. Genes regulated either on transcriptomic and/or proteomic levels were mapped against the homologous clustering approach of KpSC including *K. variicola* (KV) and *K. quasipneumoniae* (KQ) and eight different *K. pneumoniae* (KP) sequence types (STs) (sequence type (ST)11, ST15, ST23, ST101, ST147, ST258, ST307 and ST512). The regulation on transcriptomic (**A**) and proteomic (**B**) levels is shown as the log2 fold change (L2FC) of the indicted comparisons each. (**C**) The occurrence of the individual gene clusters within the different KpSC species and STs of the large clustering approach is shown in [%].

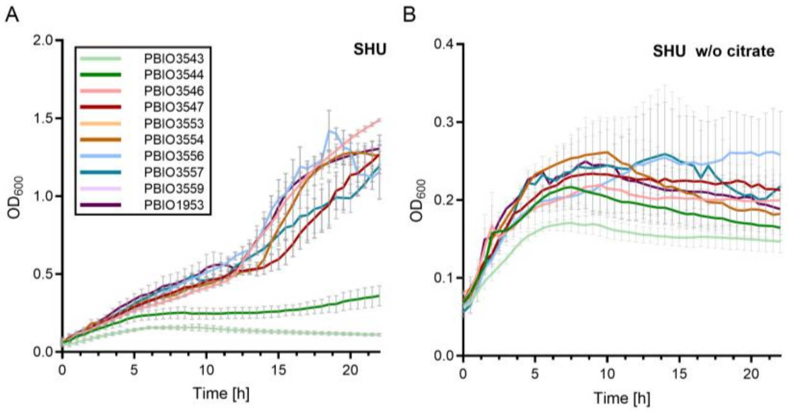

Supplementary figure 9: **Growth kinetics of *K. variicola* (KV) and *K. pneumoniae* (KP) strains in synthetic human urine (SHU) and citrate depleted SHU:** In its original recipe the SHU contains 6.8 mM citrate, which was not added to the citrate depleted SHU. Strains were grown overnight in either normal SHU or citrate depleted SHU. Cultures in a 24-well suspension plate (Sarstedt, Nümbrecht, Germany) were inoculated at an optical density of 0.05 at λ = 600 nm (OD_{600}) of 0.05 and incubated at 37 °C with double orbital shaking (180 rpm) before each measurement every 30 min using a Spark® Multimode Microplate Reader (TECAN, Switzerland). (**A+B**) Growth kinetics in either normal SHU (**A**) or citrate depleted SHU (**B**) are displayed using GraphPad Prism.
